# Sensitivity of northeastern US surface ozone predictions to the representation of atmospheric chemistry in the Community Regional Atmospheric Chemistry Multiphase Mechanism (CRACMMv1.0)

**DOI:** 10.5194/acp-23-9173-2023

**Published:** 2023-08-21

**Authors:** Bryan K. Place, William T. Hutzell, K. Wyat Appel, Sara Farrell, Lukas Valin, Benjamin N. Murphy, Karl M. Seltzer, Golam Sarwar, Christine Allen, Ivan R. Piletic, Emma L. D’Ambro, Emily Saunders, Heather Simon, Ana Torres-Vasquez, Jonathan Pleim, Rebecca H. Schwantes, Matthew M. Coggon, Lu Xu, William R. Stockwell, Havala O. T. Pye

**Affiliations:** 1 Oak Ridge Institute for Science and Engineering (ORISE), Office of Research and Development, U.S. Environmental Protection Agency, Research Triangle Park, North Carolina, USA; 2 Office of Research and Development, U.S. Environmental Protection Agency, Research Triangle Park, North Carolina, USA; 3 Office of Air and Radiation, U.S. Environmental Protection Agency, Research Triangle Park, North Carolina, USA; 4 General Dynamics Information Technology, Research Triangle Park, North Carolina, USA; 5 Office of Chemical Safety and Pollution Prevention, U.S. Environmental Protection Agency, Washington, DC, USA; 6 Chemical Sciences Laboratory, National Oceanic and Atmospheric Administration, Boulder, Colorado, USA; 7 Cooperative Institute for Research in Environmental Science (CIRES), University of Colorado Boulder, Boulder, Colorado, USA; 8 Department of Physics, University of Texas at El Paso, El Paso, Texas, USA

## Abstract

Chemical mechanisms describe how emissions of gases and particles evolve in the atmosphere and are used within chemical transport models to evaluate past, current, and future air quality. Thus, a chemical mechanism must provide robust and accurate predictions of air pollutants if it is to be considered for use by regulatory bodies. In this work, we provide an initial evaluation of the Community Regional Atmospheric Chemistry Multiphase Mechanism (CRACMMv1.0) by assessing CRACMMv1.0 predictions of surface ozone (O_3_) across the northeastern US during the summer of 2018 within the Community Multiscale Air Quality (CMAQ) modeling system. CRACMMv1.0 O_3_ predictions of hourly and maximum daily 8 h average (MDA8) ozone were lower than those estimated by the Regional Atmospheric Chemistry Mechanism with aerosol module 6 (RACM2_ae6), which better matched surface network observations in the northeastern US (RACM2_ae6 mean bias of +4.2 ppb for all hours and +4.3 ppb for MDA8; CRACMMv1.0 mean bias of +2.1 ppb for all hours and +2.7 ppb for MDA8). Box model calculations combined with results from CMAQ emission reduction simulations indicated a high sensitivity of O_3_ to compounds with biogenic sources. In addition, these calculations indicated the differences between CRACMMv1.0 and RACM2_ae6 O_3_ predictions were largely explained by updates to the inorganic rate constants (reflecting the latest assessment values) and by updates to the representation of monoterpene chemistry. Updates to other reactive organic carbon systems between RACM2_ae6 and CRACMMv1.0 also affected ozone predictions and their sensitivity to emissions. Specifically, CRACMMv1.0 benzene, toluene, and xylene chemistry led to efficient NO_*x*_ cycling such that CRACMMv1.0 predicted controlling aromatics reduces ozone without rural O_3_ disbenefits. In contrast, semivolatile and intermediate-volatility alkanes introduced in CRACMMv1.0 acted to suppress O_3_ formation across the regional background through the sequestration of nitrogen oxides (NO_*x*_) in organic nitrates. Overall, these analyses showed that the CRACMMv1.0 mechanism within the CMAQ model was able to reasonably simulate ozone concentrations in the northeastern US during the summer of 2018 with similar magnitude and diurnal variation as the current operational Carbon Bond (CB6r3_ae7) mechanism and good model performance compared to recent modeling studies in the literature.

## 1 Introduction

Both short-term acute and long-term chronic exposure to elevated surface ozone (O_3_) concentrations can be detrimental to human and ecosystem health (Bell et al., 2005; Rich et al., 2006; Larrieu et al., 2007; Iriti and Faoro, 2008; Ghosh et al., 2018; U.S. Environmental Protection Agency, 2020). The buildup of O_3_ in the lower atmosphere also has a noticeable impact on earth’s radiative budget (e.g., Brasseur et al., 1998; Stevenson et al., 2013). As a result, many countries and governments across the world have enacted legislation to limit surface ozone pollution. In the United States the current National Ambient Air Quality Standards (NAAQS) for maximum daily 8 h average ozone (MDA8 O_3_) is set at 70 parts per billion by volume (ppb) (Bachmann, 2007; U.S. Environmental Protection Agency, 2015). Despite reductions in emissions of precursor gases that lead to O_3_ formation, many areas across the US are still in non-attainment of these standards (U.S. Environmental Protection Agency, 2022a). Thus, understanding current O_3_ pollution mitigation strategies and developing new strategies for the future are pivotal if air quality standards are to be met.

The chemistry of tropospheric O_3_ formation is complex and involves the non-linear reactions of nitrogen oxides (NO_*x*_ = NO + NO_2_) with reactive organic carbon (ROC) compounds (Seinfeld and Pandis, 2006; Jacob, 1999; Heald and Kroll, 2020). Similarly, the formation of secondary fine-particle (PM_2.5_) species such as sulfate, nitrate, and secondary organic aerosol (SOA) involves complex chemistry in multiple phases and is dependent on concentrations of numerous precursor species and atmospheric oxidants. In total, this chemistry can involve thousands of individual chemical compounds and over 10 000 chemical reactions (Dodge, 2000; Stockwell et al., 2012; Jenkin et al., 2015). Due to these complex interactions as well as the role of meteorological and dry deposition processes on O_3_ and PM_2.5_ (Seinfeld and Pandis, 2006), regulatory bodies use numerical models to simulate past, current, and future (e.g., under modified emission scenarios) concentrations to inform air quality management. Rather than simulating the explicit chemistry of every known atmospheric compound and reaction, these models usually employ chemical mechanisms which simplify the atmospheric chemistry into a more limited number of species and reactions in order to capture the most important pathways for forming O_3_ and PM_2.5_ in a computationally efficient manner (Gery et al., 1989; Carter, 1990; Stockwell et al., 1997). Typically, the chemistry leading to O_3_ is represented separately from the chemistry leading to PM_2.5_ and SOA formation in chemical transport models (e.g., Pye et al., 2010; Koo et al., 2014).

The Community Multiscale Air Quality (CMAQ) model is a numerical model developed by the United States Environmental Protection Agency (U.S. EPA) to estimate O_3_, PM_2.5_, and other pollutants, both regionally in the US and in other parts of the world (http://www.epa.gov/cmaq, last access: 8 August 2023; U.S. Environmental Protection Agency, 2022b). CMAQ is available online (see “Code and data availability”) and is distributed publicly with three types of chemical mechanisms: the Regional Atmospheric Chemistry Mechanism (RACM), Carbon Bond (CB), and SAPRC. These three chemical mechanisms represent ozone chemistry with less than 1000 reactions and up to ~200 species and have been tested on multiple model domains where they show acceptable performance at estimating ambient O_3_ concentrations (e.g., Sarwar et al., 2008, 2013; Yu et al., 2010; Mathur et al., 2017; Appel et al., 2021). Currently, Carbon Bond version 6 (CB6r3 as of CMAQv5.3) is the most common mechanism used by the U.S. EPA for predicting O_3_ (Appel et al., 2021).

The Community Regional Atmospheric Chemistry Multiphase Mechanism version 1.0 (CRACMMv1.0) (Pye et al., 2023) is a next-generation chemical mechanism that was distributed for the first time with the release of CMAQv5.4 in October 2022 (U.S. EPA Office of Research and Development, 2022). CRACMMv1.0 builds on the RACM2 framework (Goliff et al., 2013) and includes new representations of several organic systems, most notably monoterpenes and aromatics, and couples gas-phase with particle-phase products. In addition, the CRACMMv1.0 mechanism provides built-in transparent mapping of emissions to mechanism species and was designed to conserve emitted carbon as well as track carbon in products as gases react and evolve. These features were included in CRACMMv1.0 to represent particulate matter formation more accurately while also maintaining the ability to predict O_3_ concentrations.

The goal of this work is to compare CRACMMv1.0 O_3_ predictions with the previously well-established RACM2 and CB6r3 chemical mechanisms and understand drivers of differences between CRACMMv1.0 and these mechanisms. Future work will present analyses evaluating CRACMMv1.0 PM_2.5_ predictions. For the comparison presented here we used the CMAQ model and performed simulations at 4 km × 4 km horizontal grid resolution for the northeastern United States (US) domain during summer 2018 (Torres-Vazquez et al., 2022). This domain was chosen specifically because areas in the northeastern US frequently violate the O_3_ NAAQS (U.S. Environmental Protection Agency, 2022a). In addition, past field studies such as the Long Island Sound Tropospheric Ozone Study (LISTOS) and future field studies (e.g., Atmospheric Emissions and Reactions Observed from Megacities to Marine Areas, AEROMMA; Warneke et al., 2022) have been designed to specifically address the issue of high O_3_ events in the New York City metropolitan area. Air Quality System (AQS) observations made during the summer of 2018 were used to aid in the evaluation. Finally, a box model was employed to study the different chemical systems and updates that were driving differences in O_3_ predictions between RACM2 and CRACMMv1.0.

## 2 Methods

### 2.1 CMAQ model

CMAQ simulations were performed for the northeastern United States (NE US) domain at 4 km × 4 km horizontal grid resolution with 35 vertical layers from 1 June through 31 August 2018 with 2 through 31 May as the simulation spinup period. In addition to CRACMMv1.0, simulations were also performed with CB6r3 using aerosol module 7 (CB6r3_ae7; AERO7) (Appel et al., 2021) and with RACM2 using aerosol module 6 (RACM2_ae6; AERO6) (Sarwar et al., 2013), both of which are available in the standard CMAQv5.3.3 release (used here) and v5.4 (latest public release). The major difference between AERO6 and AERO7 is in the representation of monoterpene SOA, with AERO7 producing more monoterpene SOA from photooxidation (Xu et al., 2018) and organic nitrates (Pye et al., 2015) than AERO6. Chemical initial and boundary conditions for the NE US domain were generated from previous nested WRF-CMAQ simulations (Weather Research and Forecasting) (12 km), which used CB6r3_ae7 (Torres-Vazquez et al., 2022). The initial and boundary conditions from CB6r3_ae7 were mapped to CRACMMv1.0 and RACM2_ae6. See the CMAQv5.4 code repository for the mapping of Carbon Bond-based mechanisms to CRACMMv1.0 for boundary and initial condition purposes. Meteorological files for the simulation were generated offline using the Weather Research Forecasting (WRF version 4.1.2) model as described by Torres-Vazquez et al. (2022), and the files were pre-processed through the Meteorology-Chemistry Interface Processor (MCIP) (Otte and Pleim, 2010) for input to the CMAQ simulations.

### 2.2 Emissions

Anthropogenic emissions were created following the 2016 version 7.2 North American Emissions Modeling Platform (Torres-Vazquez et al., 2022; U.S. Environmental Protection Agency, 2019) with updates described below. The anthropogenic emissions for CB6r3_ae7 are the same as those for the 4 km domain in the work by Torres-Vazquez et al. (2022) and include year-specific mobile emissions predicted by the MOtor Vehicle Emission Simulator (MOVES) model, airport emissions following the 2017 National Emissions Inventory (NEI) estimates from the Federal Aviation Administration (FAA) airport model, year-specific wildland fires, monitored electric generating unit (EGU) emissions, year-specific commercial marine vehicle emissions, and emissions from other sectors following the 2016v7.2 modeling platform. Primary organic aerosol (POA) in CB6r3_ae7 was considered semivolatile, and evaporated POA was allowed to undergo gas-phase reaction with OH following the work of Murphy et al. (2017). The empirical representation of anthropogenic SOA sources (potential combustion SOA, pcSOA; Murphy et al., 2017) was turned off in all cases. For a more complete description of the anthropogenic emissions employed in the CB6r3_ae7 simulations, see the work by Torres-Vazquez et al. (2022). Biogenic emissions for all mechanism simulations were calculated within CMAQv5.3.3 using the EPA’s Biogenic Emission Inventory System (BEIS v3.6.1) (Bash et al., 2016).

CRACMMv1.0 emission inputs build on the same methods as the CB6r3_ae7 inputs with a few additional updates. The total mass and speciation of emissions from volatile chemical products were updated to follow VCPy, a model for predicting volatile chemical product (VCP) emissions (Seltzer et al., 2021). Individual ROC species were mapped to CRACMMv1.0 species as described by Pye et al. (2023). Primary organic aerosol in CRACMMv1.0 was considered semivolatile with volatility profiles of alkane-like emissions for diesel vehicles, gasoline vehicles, and aircraft (Lu et al., 2020) and slightly oxygenated species profiles for biomass burning and all other POA sources. For sources without specific volatility profiles, the volatility profile of meat-cooking emissions was used to produce a lower bound on the evaporation of semivolatile species (Woody et al., 2016; Mohr et al., 2009). Semivolatile POA was implemented using the Detailed Emissions Scaling, Isolation, and Diagnostic (DESID) module in all cases (Murphy et al., 2021).

The anthropogenic emissions created for CRACMMv1.0 were also used with slight adjustments for RACM2_ae6 simulations in CMAQ (see Table S1 in the Supplement for mappings). For the RACM2_ae6 simulations, primary organic aerosol (POA) was treated as semivolatile with the same volatility profiles as in the CRACMMv1.0 simulations but with the chemistry of AERO6 (Murphy et al., 2017). Alkane-like semivolatile and intermediate-volatility organic compounds (S/IVOCs) emitted in the gas phase were ignored in RACM2_ae6, and the empirical representation of anthropogenic SOA sources (pcSOA, Murphy et al., 2017) was turned off in RACM2_ae6 as in CB6r3_ae7.

### 2.3 Air quality network observations

Surface-level network observations of air pollutants made in the northeastern US between June and August 2018 were used to evaluate CMAQ model outputs. Hourly measurements of O_3_ and NO_*x*_ were obtained from the AQS database using the available pre-generated files and paired in time and space with model quantities using the Atmospheric Model Evaluation Tool (AMET) (Appel et al., 2011). The observations in AQS were quality-assured by the reporting agency (e.g., EPA, states, tribes), and therefore no additional quality checks of AQS data were done in AMET. In the case of time periods with missing data, those missing periods were removed from the analysis. In cases where multiple observations were reported for a single site using different parameter occurrence codes (POCs), those observations were treated as individual measurements with the POC number used to distinguish between the different measurements for the same site.

### 2.4 Box modeling in F0AM

The Framework for 0-D Atmospheric Modeling (F0AMv4.2) box model was used as a tool to examine differences in chemistry between the mechanisms (Wolfe et al., 2016). Chemical species and reactions from the RACM2 and CRACMMv1.0 mechanisms were ported into F0AM from CMAQ-ready mechanism files using a custom MATLAB script (see “Code and data availability”). Photolysis rates in RACM2 and CRACMMv1.0 were matched to existing Master Chemical Mechanism (MCM) rates in F0AM, and the F0AM default example actinic flux rates were prescribed for all simulations. Three chamber experiments were run by initiating experiments with 10 ppb of either *α*-pinene, isoprene, or benzene under high- (5 ppb) and low-NO_*x*_ (0.5 ppb) conditions at standard temperature (*T* = 298 K) and pressure (*P* = 1013 mbar). Hydrogen peroxide, set at 200 ppb, was used as the radical OH source (~ 2 × 10^−4^ ppb initial OH), and relative humidity was set at 10 % across all simulations. After initiation, each chemical system was allowed to evolve for 24 h to reach steady state before the simulation was terminated. In addition, to gain insight into the role organic vs. inorganic updates played in O_3_ production in CRACMMv1.0, all three ROC precursors were re-run in simulations using a modified RACM2 mechanism (RACM2_mod) where all inorganic rate constants in RACM2 were updated to match those in CRACMMv1.0. This was needed because the development of CRACMMv1.0 not only incorporated updates to various ROC reaction systems in terms of product yields and chemical fates but also included inorganic rate constant updates (> 20 rate constants) to reflect current literature values, which differ from those prescribed in RACM2.

## 3 Ozone predictions

### 3.1 Ozone predictions by mechanism

**Figure 1.**
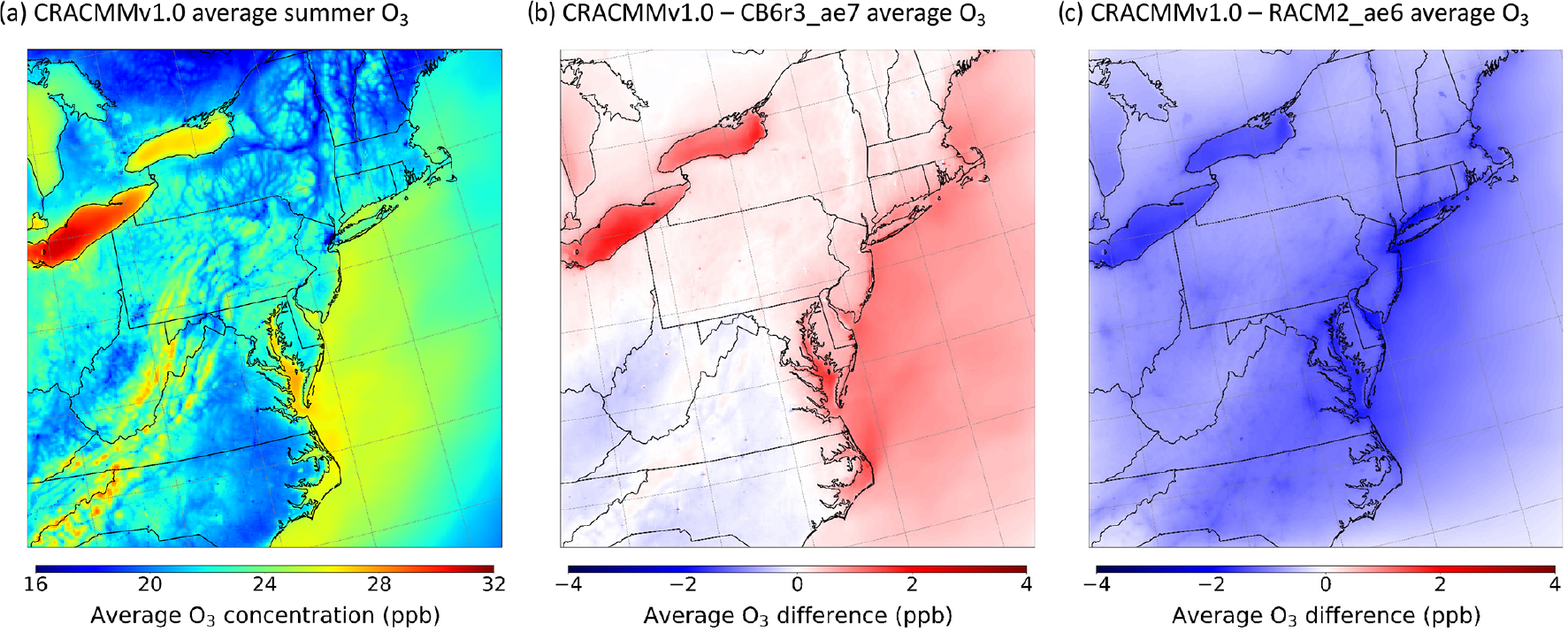
**(a)** Simulated summer (June–August) 2018 surface ozone average (all hours) as predicted by CRACMMv1.0. Simulated summer ozone average (all hours) differences of **(b)** CRACMMv1.0 − CB6r3_ae7 and **(c)** CRACMMv1.0 − RACM2_ae6.

Figure 1a shows the June–August average surface ozone concentration (averaged for all hours) predicted by the CRACMMv1.0 chemical mechanism across the northeastern US model domain. CRACMMv1.0 average ozone predictions ranged from 16–32 parts per billion by volume (ppb), with the highest average ozone predictions occurring over the Great Lakes region, Appalachian Mountain region, and the Atlantic coastline. The higher average O_3_ predictions (28–32 ppb) in the Great Lakes region and around Chesapeake Bay (Fig. 1) have been shown to be driven by land–water circulation due to the difference in daytime planetary boundary layer (PBL) heights over cool water (typically < 300 m) compared to much higher PBL heights over land (often 1500–2500 m) (Dye et al., 1995; Lennartson and Schwartz, 2002; Foley et al., 2011; Dreessen et al., 2019; Cleary et al., 2022). In particular, O_3_ exceedance events around Lake Michigan have been predominantly attributed to the northeasterly transport of O_3_ and O_3_ precursors to the lake, where photochemical O_3_ production then becomes intensified under conditions of lower vertical mixing and lower dry deposition (Sillman et al., 1993; Dye et al., 1995; Lennartson and Schwartz, 2002; Foley et al., 2011; Cleary et al., 2022). These lake effects often lead to regular NAAQS exceedances in the region (Foley et al., 2011). The elevated O_3_ concentrations predicted for the Appalachian Mountain region have also been shown to be driven primarily by the transport of O_3_ and other pollutants from nearby urban centers and coal-fired power plants (Aneja et al., 1991; Neufeld et al., 2019). In addition, O_3_ losses in the region have been measured to be lower at the higher elevation on the mountaintops, which leads to the buildup of O_3_ during the night (Aneja et al., 1991; Neufeld et al., 2019).

The magnitude of the ozone concentrations predicted by CRACMMv1.0 was in good agreement with O_3_ predictions from the base CB6r3_ae7 simulation, with inland differences typically falling below ±1 ppb across the model domain (Fig. 1b). These absolute differences corresponded to relative differences of ±5 % (Fig. S1a). The largest observed spatial discrepancies between the two mechanisms occurred near bodies of water, where CRACMMv1.0-estimated average ozone was ~ 2–4 ppb higher than estimates made by the CB6r3_ae7 chemical mechanism. The higher predicted differences near water are likely explained by intensified chemistry due to the land–water circulation effect described previously, which generally drives the higher O_3_ concentrations in the regions. In addition, Foley et al. (2011) and Vermeuel et al. (2019) found that O_3_ production showed greater NO_*x*_ sensitivity as urban plumes advected across Lake Michigan. Thus, differences in O_3_ production near waterbodies between the simulations were influenced by the representation of O_3_–NO_*x*_–ROC chemistry in the two mechanisms and their characterization of the chemical regime. Differences in chemical production of O_3_ between CRACMMv1.0 and CB6r3_ae7 are discussed and further explored later (Sect. 4.2). The differences over water between CB6r3_ae7 and CRACMMv1.0 were not expected to be driven by dry deposition over the Great Lakes as deposition is largely suppressed over water (Sillman et al., 1993).

Because different VCP emission inventories were employed between the CRACMMv1.0 and CB6r3_ae7 simulations (see Sect. 2.2), differences in the two inventory methods, in addition to differences in chemistry, could account for a small fraction of the differences shown in Fig. 1b. This would be expected to have the most pronounced effect over urban areas, where VCP emissions are largest. In a previous model study, simulations showed that a complete removal of VCP emissions led to a 1 ppb O_3_ change in downtown New York City over a 24 h period (Seltzer et al., 2022); thus, the choice of VCP inventory is expected to result in differences much less than 1 ppb.

In comparison with RACM2_ae6, CRACMMv1.0 estimated a lower average concentration (average O_3_ difference of 2–4 ppb) across the model domain, with the largest differences in predictions occurring near urban centers in the metropolitan northeastern US in addition to coastal areas along the Great Lakes region and the Atlantic seaboard (Fig. 1c). The mechanism-to-mechanism average O_3_ differences presented in Fig. 1c corresponded to relative average O_3_ differences of 0 %–15 % between the mechanisms across the model domain (Fig. S1b). The coupling of meteorology and chemistry, similar to the situation discussed for Lake Michigan, could again explain the larger relative differences in O_3_ concentrations near waterbodies (Fig. 1c). Since RACM2_ae6 emissions were mapped from CRACMMv1.0 inputs, the differences between these simulations were due to chemical differences between the mechanisms alone. Over land, differences in O_3_ predictions between CRACMMv1.0 and RACM2_ae6 were smaller (< 2 ppb, < 7 %) but were consistently biased in one direction (Figs. 1c, S1b). These findings suggest that updates in chemistry between RACM2_ae6 and CRACMMv1.0 led to a ubiquitous reduction in O_3_ across the model domain. The role of chemistry as a driver in mechanism-to-mechanism ozone differences between RACM2_ae6 and CRACMMv1.0 is revisited in Sect. 4.

### 3.2 Evaluation of spatial distribution

**Figure 2.**
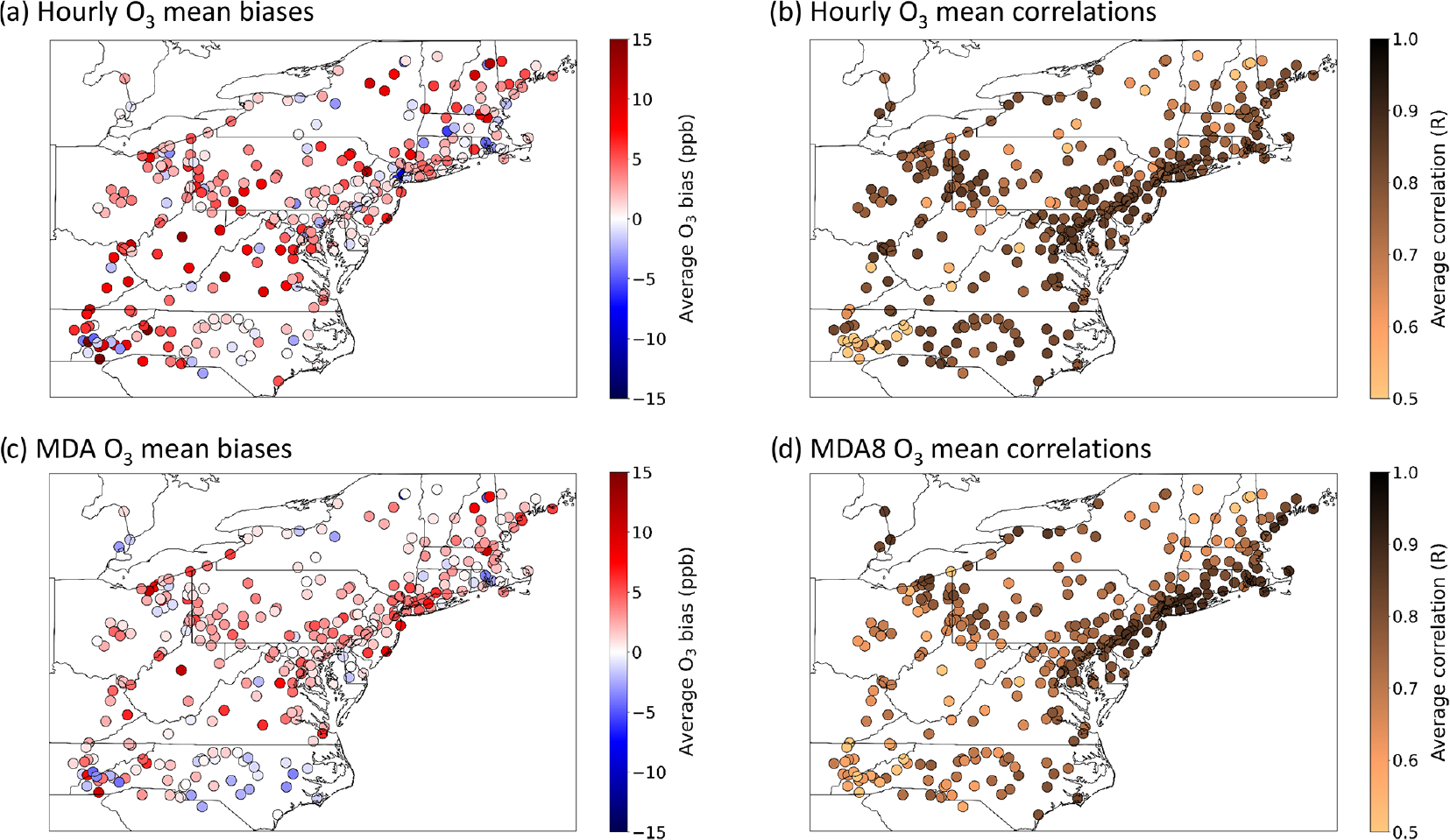
Ozone **(a, c)** mean biases (in ppb) and **(b, d)** correlations between predictions and observations for **(a–b)** all hourly O3 values and **(c–d)** MDA8 O3 values across the NE US for CRACMMv1.0 calculated using AQS observations between June–August 2018.

Hourly ozone performance statistics were calculated by pairing CMAQ outputs in space and time with 313 AQS sites that reported hourly observations between the months of June and August 2018 using AMET (See Sect. 2.3). Figure 2a and b show the spatial distribution in model–observation hourly mean biases and linear correlations (*r*) between predictions and observations for all hourly observations covered by the CRACMMv1.0 simulation. In general, the hourly O_3_ mean bias (MB) indicates a high bias across the model domain, with the highest biases (> 15 ppb) occurring along the North Carolina–Tennessee border (Fig. 2a). Model biases were much lower around the metropolitan NE US (Washington, DC; Maryland; New Jersey; New York City–Long Island regions), where predictions fell within ±4 ppb of the observed average values. Linear correlations between hourly O_3_ estimates and observations at a given AQS site were typically high (*r* > 0.8) in the northeastern US (Fig. 2b). Correlations between hourly observations and predictions were the weakest at sites located in the Appalachian Mountain region (*r* = 0.4–0.6) and were strongest at sites located in the metropolitan northeastern US (*r* > 0.9). The hourly O_3_ normalized mean bias (NMB) and normalized mean error (NME) across the domain can be found in the Supplement (Fig. S2), and values followed a similar spatial distribution as Fig. 2a and b, with lower NMB (−20 % to +20 %) and NME (< 30 %) values nearer population centers (e.g., Washington, DC; Baltimore; Philadelphia; Boston) and higher NMB (+ 20 %–100 %) and NME (> 40 %) at sites further from city centers (Fig. S2). Even so, hourly ozone predictions had values of NMB between ±20 % at 250 out of 313 sites and NME less than 30 % at 227 out of 313 of the reporting sites.

The bias and correlation for daily maximum 8 h average ozone concentration (MDA8 O_3_) were also calculated for CRACMMv1.0 at each site (Fig. 2c, d). Predictions of MDA8 O_3_ are often used by regulating bodies, such as the U.S. EPA, to determine whether regions are in attainment or non-attainment of national ozone air quality standards. Predictions of MDA8 O_3_ also reflect a model’s ability to estimate daytime O_3_ concentrations as O_3_ concentrations are higher during the day. CRACMMv1.0 MDA8 O_3_ mean biases were similar to the reported hourly O_3_ biases and ranged from −4 to +16 ppb across the model domain, with model–observation biases falling within ±4 ppb at 245 out of 313 sites (Fig. 2c). Correlations between modeled and observed MDA8 O_3_ were also determined to be high (Fig. 2d), and CRACMMv1.0 MDA8 O_3_ predictions showed a stronger correlation than hourly O_3_ predictions at the Appalachian Mountain sites (e.g., North Carolina–Tennessee border) but were weaker in central North Carolina and in Ohio. MDA8 O_3_ normalized mean biases did not exceed ±40 %, with 305 sites reporting normalized mean biases within ±20 % (Fig. S2c). MDA8 O_3_ normalized mean errors did not exceed 45 % across the domain, and NME values were lower than 20 % for the majority (95 %) of sites (Fig. S2d).

Hourly and MDA8 O_3_ predictions that were biased high were not isolated to the CRACMMv1.0 simulation as both the CB6r3_ae7 and RACM2_ae6 hourly and MDA8 O_3_ estimates showed high biases over the northeastern US in summer 2018 (Figs. S3–S6). High summer O_3_ daytime and nighttime biases have been noted in previous studies in CMAQ investigating air quality over the northeastern US and contiguous US (CONUS) using the RACM2 and CB6 mechanisms (Appel et al., 2021; Sarwar et al., 2013; Cheng et al., 2022). Cheng et al. (2022) noted in their study that daytime high O_3_ biases were reduced by a more accurate representation of cloud cover via the assimilation of satellite data. Nighttime overestimation of O_3_ in a previous study using CMAQ, on the other hand, was attributed to high O_3_ coming in from the domain boundaries and low vertical mixing (Li and Rappenglueck, 2018). The exact drivers of the high summer O_3_ estimates in CMAQ, however, are still under investigation. The calculated hourly and MDA8 ozone statistics for the CB6r3_ae7 and RACM2_ae6 simulations were found to be of very similar spatial distribution and magnitude to those calculated for CRACMMv1.0 (Figs. 2 and S2–S6), where both simulations reported lower biases in the metropolitan NE US and higher biases in other areas of the domain. Given that all mechanism O_3_ biases were lowest nearer to major cities, this suggests that the CMAQ simulations better estimated O_3_ concentrations in areas exposed to higher levels of anthropogenic pollutants.

Table 1 summarizes the domain-wide averages of site-specific ozone performance statistics for all three mechanisms and highlights that CRACMMv1.0 performed well when compared with domain-wide hourly and MDA8 O_3_ estimates from RACM2_ae6 and CB6r3_ae7. The lower O_3_ estimates by CB6r3_ae7 across the domain most closely matched observations and showed the lowest domain-wide hourly and MDA8 O_3_ mean bias (MB), normalized mean bias (NMB), and normalized mean error (NME). CRACMMv1.0 hourly O_3_ predictions showed a similar MB (+2.7 ppb vs. +2.4 ppb) and NMB (+8.8 % vs. +7.9 %) to CB6r3_ae7, while CRACMMv1.0 MDA8 O_3_ MB (+2.1 ppb vs. +1.5 ppb) and NMB (+7.7 % vs. +3.4 %) values were slightly higher than CB6r3_ae7.

While on average, hourly O_3_ and MDA8 O_3_ were slightly overestimated by all mechanisms, the highest O_3_ values were generally underestimated by all mechanisms (Table 1). For the subset of conditions where observed O_3_ was above 50 ppb (approximately the highest 10 % of concentrations) RACM2_ae6 (MB of −1.7 ppb) performed best followed by CRACMMv1.0 (MB of −4.7 ppb) and then CB6r3_ae7 (MB of −6.2 ppb). CRACMMv1.0 with the Automated MOdel REduction (AMORE) representation of isoprene chemistry (CRACMM1AMORE) is expected to perform even better than CRACMMv1.0 at high ozone concentrations (Wiser et al., 2022).

Emery et al. (2017) characterized NMB and NME model statistics from modeling studies reported in the literature (Simon et al., 2012) and found that two-thirds of modeling studies reported hourly and MDA8 NMB of < 15 %, NME of < 25 %, and *r* of > 0.50. With the exception of domain-wide hourly O_3_ NME, all mechanisms examined here had model performance (NMB, NME, and *r*) within the range reported in the literature. By these metrics, CRACMMv1.0 performs consistently with state-of-science criteria for predicting O_3_ in photochemical models while also treating the loss of mass to SOA formation.

### 3.3 Evaluation of diurnal distribution

**Figure 3.**
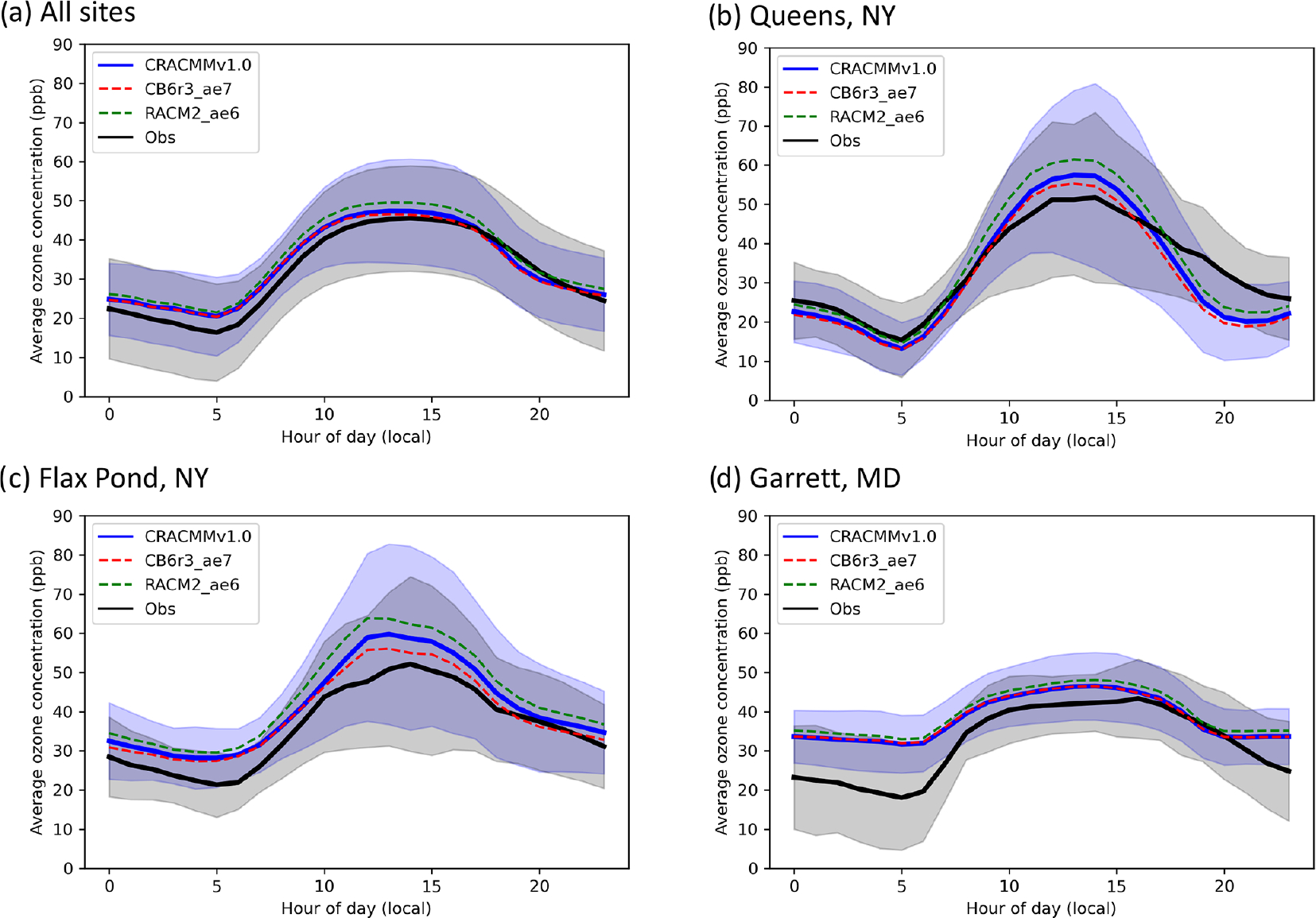
Average (± standard deviation) hourly O3 concentrations predicted by CMAQ using CRACMMv1.0 (blue trace) and observed (black trace) at **(a)** all AQS sites within the domain; **(b)** Queens, NY (AQS site 36-081-0124); **(c)** Flax Pond, NY (AQS site 36-103-0044); and **(d)** Garrett, MD (AQS site 24-023-0002) during June, July, and August 2018. Predicted average hourly O3 values in the CB6r3_ae7 CMAQ simulation (dashed red trace) and the RACM2_ae6 CMAQ simulation (dashed green trace) are also overlaid in each panel.

Figure 3a shows the diurnal average hourly ozone surface concentrations (±1 standard deviation) estimated by CRACMMv1.0 (blue trace) compared to average hourly network observations (±1 standard deviation) for all AQS sites (black trace) that reported measurements during the summer of 2018 within the domain. Figure 3a shows that CRACMMv1.0 captured the general diurnal pattern of the observed ozone concentrations across the model domain, and predictions fell within the standard deviation of the observations. CMAQ simulations using CRACMMv1.0 predicted a similar onset in O_3_ production and an earlier and sharper decline in afternoon O_3_ than what was typically observed at the AQS sites. The model also predicted higher average nighttime minimum O_3_ than what was observed. The average summer diurnal O_3_ concentrations predicted by CMAQ using the CB6r3_ae7 (dashed red trace) and RACM2_ae6 (dashed green trace) mechanisms followed the same diurnal trend, with CRACMMv1.0 and CB6r3_ae7 simulations showing better agreement with hourly observations than the RACM2_ae6 simulation (Fig. 3a).

Because the offset observed in morning growth and late-afternoon decline in O_3_ between CMAQ and the AQS observations was predicted by all mechanism simulations, meteorology was likely a driving contributor to the model–observation discrepancies during these time periods. For example, a previous study comparing CMAQ O_3_ predictions across North America determined that the timing of the diurnal ozone signal was likely driven by boundary layer dynamics in the model over emissions or chemistry (Solazzo et al., 2017). As mentioned in Sect. 3.2 the high nighttime biases observed in Fig. 3a could have also been driven by meteorology or by O_3_ coming in from the boundaries (Li and Rappenglueck, 2018). However, mechanism-to-mechanism differences and, more specifically, predictions of peak O_3_ during the daytime are influenced by the different treatments of chemistry between the simulations.

To further examine how different treatments of chemistry and/or emissions impacted hourly O_3_ differences between mechanisms compared to observations, comparisons at three selected AQS sites (one urban, one suburban, and one rural site) were also plotted in Fig. 3b, c, and d. Queens, NY, was chosen as a representative urban site (average hourly [NO_*x*_]_mod_ ≈ 12 ppb); Flax Pond, NY, was chosen as a representative suburban site (average hourly [NO_*x*_]_mod_ ≈ 3 ppb); and Garrett, MD, was chosen as a representative rural/remote site (average hourly [NO_*x*_]_mod_ < 1 ppb). Similar to Fig. 3a, all mechanism predictions fell within the standard deviation of the observations at all hours for all three sites (Fig. 3b, c, d). The RACM2_ae6 simulation showed the greatest diurnal change in hourly O_3_ concentrations (daytime ozone production) and highest daytime biases, while CB6r3_ae7 predicted the smallest changes in hourly O_3_ (daytime ozone production) and showed the lowest daytime biases at all three sites. All simulations showed the lowest hourly relative biases (±10 %) at the urban site (Queens, NY), suggesting that the model provides reasonable prediction of O_3_ production under high-NO_*x*_ conditions. This reduced bias in an urban area is consistent with the hourly O_3_ biases shown previously across the northeastern US (Figs. 2 and S2–S6), where spatial biases were found to be lowest in the metropolitan NE US where local ozone formation is expected to make up a larger fraction of total ozone than at more rural locations. Larger differences between hourly mechanism-to-mechanism O_3_ predictions were observed at the more polluted sites. In particular, the daytime O_3_ estimated by RACM2_ae6 at Queens and Flax Pond (Fig. 3b, c) showed a much larger relative increase to CRACMMv1.0 and CB6r3_ae7 than what was seen at Garrett, MD (Fig. 3d). Again, this may in part be due to larger relative contribution from boundary conditions and transported ozone at rural locations vs. urban locations. Modeled NO_*x*_ concentrations at all the sites were similar between mechanisms (within ±0.05 ppb), and the relationship between ozone production and NO_*x*_ is further explored in the following section.

## 4 Drivers of ozone formation

In this section, CMAQ simulations with emission perturbations are combined with box modeling to understand drivers of ozone formation. In addition, mechanism ozone production efficiency is quantified using modeled NO_*x*_ and O_3_ concentrations across the northeastern US.

### 4.1 Sensitivity to specific ROC emissions

**Figure 4.**
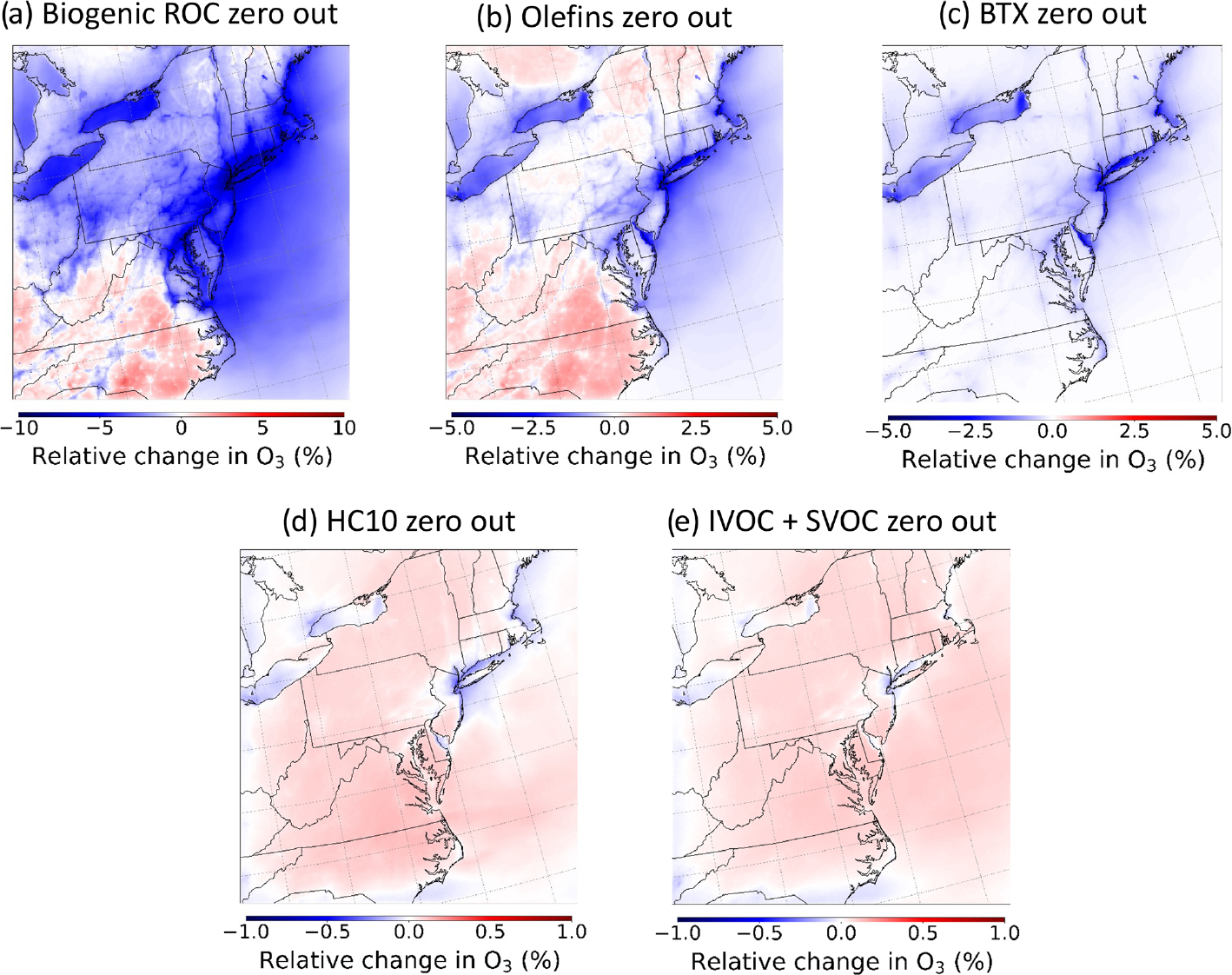
Relative changes in O3 concentrations from the CRACMMv1.0 base simulation (zeroed emissions – base) for the **(a)** zeroed biogenic-emission scenario, **(b)** zeroed olefin emission scenario, **(c)** zeroed BTX emission scenario, **(d)** zeroed HC10 emission scenario, and **(e)** zeroed S/IVOC emission scenario.

A series of emission sensitivity simulations were performed in CMAQ to gain insight into the precursor ROC systems important for O_3_ formation in CRACMMv1.0 across the NE US summer 2018 model domain. The sensitivity simulations were conducted by running a set of zeroed emission simulations (i.e., setting emissions of a chemical class or emission sector to zero) and determining the response in O_3_ concentrations to the emission perturbation. A list of all the zeroed emission simulations can be found in Table 2. Due to the non-linear response of ozone production to perturbations in NO_*x*_ concentrations, the interpretations of zeroed emission simulations can be challenging. Nonetheless, these types of perturbations provide an initial assessment of the ozone production response in CRACMMv1.0 and provide insight into how chemical systems respond to lower ROC emissions in CRACMMv1.0 vs. RACM2_ae6 and CB6r3_ae7. Figure 4 shows domain-wide percent differences in average ozone concentrations (ΔO_3_) between the base CRACMMv1.0 simulation and a series of zeroed emission simulations. The largest ΔO_3_ response occurred when emissions from biogenic sources were excluded from the simulation (Fig. 4a). The zeroed biogenic-emission simulation resulted in percent changes in average O_3_ concentrations ranging from −10 % to +3 %. Spatially, average O_3_ concentrations decreased by ~ 5 %–10 % in the metropolitan northeastern US and increased in the southern part of the model domain in response to the perturbation. Relatively large changes in ΔO_3_ were also predicted in the zeroed olefin and benzene–toluene–xylene (BTX) emission simulations, with average O_3_ concentration changes ranging from −4 % to +2 % (Fig. 4b, c). A similar spatial response in ΔO_3_ was seen between the zeroed biogenic-emission and anthropogenic-olefin emission simulations (Fig. 4a, b), while the response of ΔO_3_ in the zeroed BTX emission simulation was localized to urban areas, particularly in the metropolitan NE US and never indicated disbenefits (Fig. 4c). The chemical formation of O_3_ in CRACMMv1.0 was less sensitive to large alkanes (HC10) and semivolatile and intermediate-volatility organic compound (S/IVOC) emissions across the model domain as a ΔO_3_ response of +1 % was predicted in these simulations (Fig. 4d, e). All five sensitivity simulations showed some reduction in O_3_ in the New York City urban core with ROC reductions indicating ROC-sensitive ozone formation.

A ΔO_3_ response like the one in CRACMMv1.0 was also predicted when biogenic emissions were zeroed in a simulation run with RACM2_ae6 (+3 % to −10 %) (Fig. S7), indicating that biogenic emissions were important to O_3_ formation across chemical mechanisms in the northeastern US domain. This strong sensitivity of O_3_ formation to biogenic-ROC emissions in the eastern and northeastern United States has also been noted in previous chemical transport model studies (e.g., Hogrefe et al., 2004; Fiore et al., 2005). A slightly higher and more widespread decrease in ΔO_3_ was seen in the RACM2_ae6 zeroed biogenic-emission simulation (Fig. S7) than in the CRACMMv1.0 zeroed emission simulation (Fig. 4a), which suggests different representations of biogenic-ROC chemistry between CRACMMv1.0 and RACM2_ae6 lead to some of the differences in modeled O_3_ concentration shown in Figs. 2 and 4. Zeroed BTX emission simulations run using RACM2_ae6 and CB6r3_ae7 (Figs. S8 and S9) resulted in ΔO_3_ responses (−2 % to −4 %) around urban areas similar to those that were observed in the CRACMMv1.0 zeroed BTX emission simulation (Fig. 4c). Domain-wide BTX emission effects on ozone were lower than biogenic-emission effects and more pronounced in urban source regions. Unlike CRACMMv1.0, the RACM2_ae6 and CB6r3_ae7 simulations predicted slightly higher ozone concentrations (ΔO_3_ = +1 %) in non-urban regions in the domain in the zeroed BTX emission simulations compared to the base model run (Figs. S8 and S9). Note that the organic nitrate yield in aromatic systems was reduced from 8.2 % to 0.2 % based on recent work by Xu et al. (2020) in CRACMMv1.0 (Pye et al., 2023). This change increases NO-to-NO_2_ conversion, which indicates BTX oxidation generally leads to ozone production in CRACMMv1.0. However, CRACMMv1.0 also removes radicals from the gas phase when autoxidation or phenol chemistry leads to SOA, thus reducing radical abundances, and Sect. 4.2 will illustrate that CRACMMv1.0 has a different baseline O_3_ prediction than RACM2_ae6 for benzene. These results indicate that the differing representation of aromatic chemical systems within the mechanisms explains some of the differences in modeled O_3_ concentrations shown in Sect. 3.

The modeled reductions in O_3_ seen near urban regions (Fig. 4a–c) and in the New York City urban core specifically (Fig. 4a–e) are mechanistically consistent for regions expected to have relatively high emissions of NO_*x*_, and thus reductions in ROC would lead to less ozone production. In these more ROC-sensitive regions, ozone production drops due to changes in total ROC reactivity. When ROC emission reductions are large enough (such as in the zeroed biogenic-ROC emission simulation in Fig. 4a), even NO_*x*_-sensitive locations could transition to a NO_*x*_-saturated chemical regime, where ROC reductions reduce ozone. The zeroed emission simulations often showed less sensitivity in the ΔO_3_ response to emission reductions in rural/remote regions (Fig. 4a–c) and even predicted an increase in O_3_ formation in rural regions in response to some emission perturbations (Fig. 4a, b, d, e). S/IVOCs and large alkanes (HC10) in particular suppressed ozone formation in the base simulation as indicated by their zeroed emission simulations, leading to increases in ozone with the exception of the New York City urban core (Fig. 4d, e). The ozone formation potential for HC10 compounds across the entire US for all of 2017 was high in previous work due to the overall abundance of emissions despite low maximum incremental reactivity (MIR) (Pye et al., 2023); however a much smaller change in average O_3_ concentration (±1 %) was observed in the zeroed HC10 emission simulation here compared to the olefin and BTX simulations. This result suggests that the emissions of HC10 compounds were relatively less important to ozone formation in the NE US domain compared to the entire US for all of 2017. Given the low MIR of IVOC and SVOC compounds, zeroing the emissions of these compounds was expected to have mild impacts on O_3_ formation, and Fig. 4e showed that O_3_ concentrations increased by ~ 0.5 % across the full domain.

The emission perturbation results suggest that large volatile alkanes (HC10 and S/IVOCs) primarily act to sequester oxidants such as OH and NO_*x*_, thus resulting in increases in O_3_ for the zeroed emission simulations. Specifically, S/IVOC alkanes as well as HC10 in CRACMMv1.0 sequester NO_*x*_ with the high efficiency due to a 26 %–28 % yield of alkyl nitrates (Pye et al., 2023). This hypothesis is supported by observed domain-wide increases (up to 4 %) in NO_2_ when both HC10 and SVOC emissions are removed from the simulations (Figs. S10 and S11). In addition, organic nitrates decrease up to 10 % near the urban core when HC10 emissions are omitted from the simulation (Fig. S12). Decreases in organic nitrate formation due to emission removal could also explain the increases in O_3_ formation seen in the rural regions of the zeroed biogenic-emission and olefin emission simulations (Fig. 4a, b), where O_3_ formation would increase in response to less NO_*x*_ loss in a NO_*x*_-sensitive regime.

### 4.2 Ozone production efficiency

**Figure 5.**
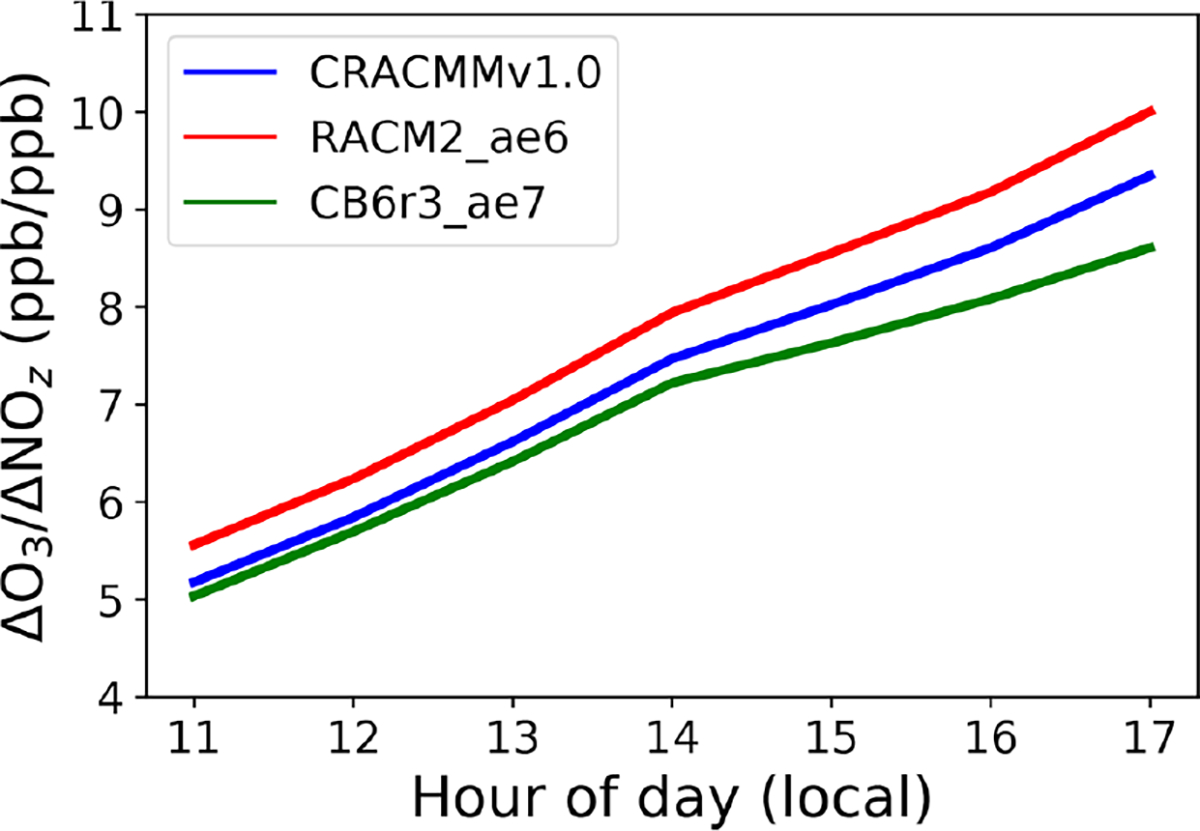
Average domain-wide hourly ozone production efficiency (OPE) calculated from the slope of the linear regression between NO*z* and O3 at a given hour between 11:00 and 17:00 local time for the CRACMMv1.0, RACM2_ae6, and CB6r3_ae7 mechanism base simulations.

Ozone production efficiency (OPE) is defined as the number of molecules of O_3_ produced per molecule of NO_*x*_ loss and can be viewed as a metric describing chain length in O_3_ propagation before NO_*x*_ is chemically removed from the atmosphere (Jacob, 1999). Thus, model-constrained OPE estimates can provide mechanistic insight into O_3_–NO_*x*_–ROC cycling within a given chemical system or region. Operationally, OPE has been calculated using the slope of the linear regression between O_3_ and the sum of all NO_*x*_ oxidation products (NO_*z*_) as O_3_ and NO_*z*_ evolve during the photochemically active hours of the day (e.g., Arnold et al., 2003; Sarwar et al., 2013; Henneman et al., 2017). This OPE proxy (ΔO_3_ /ΔNO_*z*_) provides a good first-order approximation of OPE but may not sufficiently capture ozone recycling in regions impacted by fresh NO_*x*_ emissions and regions where NO_*x*_ and NO_*z*_ losses through deposition are high. Using this proxy (i.e., ΔO_3_ /ΔNO_*z*_) we estimated mechanism domain-wide OPE values for the northeastern US (Fig. 5). This calculation leveraged the fact that different locations experienced air masses of different ages and ΔO_3_ /ΔNO_*z*_ can be calculated using the linear relationship between O_3_ and NO_*z*_ concentrations across all grid cells in the model domain for each hour of the day. The OPE proxy showed very strong linear correlations between O_3_ and NO_*z*_ (*r* > 0.7) between the hours of 11:00 and 17:00 local time. The ΔO_3_ /ΔNO_*z*_ values showed a linear increase from the morning to the evening for all three mechanisms and were consistently highest for the RACM2_ae6 simulation and consistently lowest for the CB6r3_ae7 mechanism for all hours of the day. The OPE values evolved at similar rates during the day between the three mechanisms and reached a peak between the hours of 16:00 and 17:00 local time (Fig. 5).

Figure 5 indicates that there are either differences in O_3_ production or NO_*x*_ recycling or a combination of both between mechanisms and that these differences persist at all hours during the day. The trend in OPE values (CB6r3_ae7 < CRACMMv1.0 < RACM2_ae6) is consistent with the diurnal trends in the modeled O_3_ concentrations observed in Fig. 3. This trend in mechanisms was noted in a previous study model where RACM2_ae6 OPE predictions were shown to be consistently higher than Carbon Bond version 5 (specifically CB05TUCL) OPE predictions, leading to a poorer match with observations than Carbon Bond in the southeastern US (Sarwar et al., 2013). Figure 5 confirms that updates between RACM2_ae6 and CRACMMv1.0 led to decreases in OPE and improvement in CRACMM O_3_ predictions with observations in the northeastern US (Fig. 2; Table 1). In the following section, differences in the representation of chemical systems between RACM2_ae6 and CRACMMv1.0 that may have led to differences in ozone production and/or NO_*x*_ loss between the two mechanisms are further explored.

### 4.3 Box model simulations

The F0AM box model (Wolfe et al., 2016) was used to further probe the mechanistic drivers of differences between the CRACMMv1.0 and RACM2 chemical mechanisms that could be important for photochemical O_3_ production. Note that, for this study, only the gas-phase aspects of the RACM2 base mechanism from CMAQ were ported and tested in F0AM; thus, RACM2 rather than RACM2_ae6 nomenclature will be used to refer to these results throughout this section. The box model investigation focused on RACM2 and CRACMMv1.0 because the definitions of chemical species and ROC families are similar between mechanisms, allowing for a more direct chemical comparison between the mechanisms. In addition, CRACMMv1.0 was built upon the RACM2 framework and can be more incrementally tested. Differences in chemistry between Carbon Bond- and RACM-based mechanisms have been explored previously (Sarwar et al., 2013), and detailed analyses are beyond the scope of this study.

Box model simulations were initiated in batch mode with 10 ppb of a precursor ROC, 200 ppb of H_2_O_2_ (OH source), and either 5 ppb of NO_2_ (NO_*x*_ conditions typically observed at the Queens, NY, and Flax pond, NY, sites from Fig. 3) or 0.5 ppb NO_2_ (NO_*x*_ conditions typically observed at the Garrett, MD, site from Fig. 3). The chemical systems were allowed to evolve for 24 h to reach steady state (see Sect. 2.5 for a full description of the model setup). The dominant fate of RO_2_ in simulations under high-NO_*x*_ conditions was confirmed to be RO_2_ + NO, while simulations initiated with NO_*x*_ concentrations of 0.5 ppb were dominated by RO_2_ + RO_2_ reactions. For each simulation, the evolution of O_3_ was monitored over time. Box model simulations were run with *α*-pinene, isoprene, and benzene as the ROC precursors because the *α*-pinene and aromatic chemical systems underwent major updates in CRACMM compared to RACM2. Additionally, the CRACMMv1.0 and RACM2 zeroed biogenic-emission and BTX emission simulations (Figs. 4 and S7–S9) showed substantial impact on ambient O_3_ concentration (anthropogenic-olefin chemistry, although important for O_3_ formation, remained unchanged between RACM2 and CRACMMv1.0).

**Figure 6.**
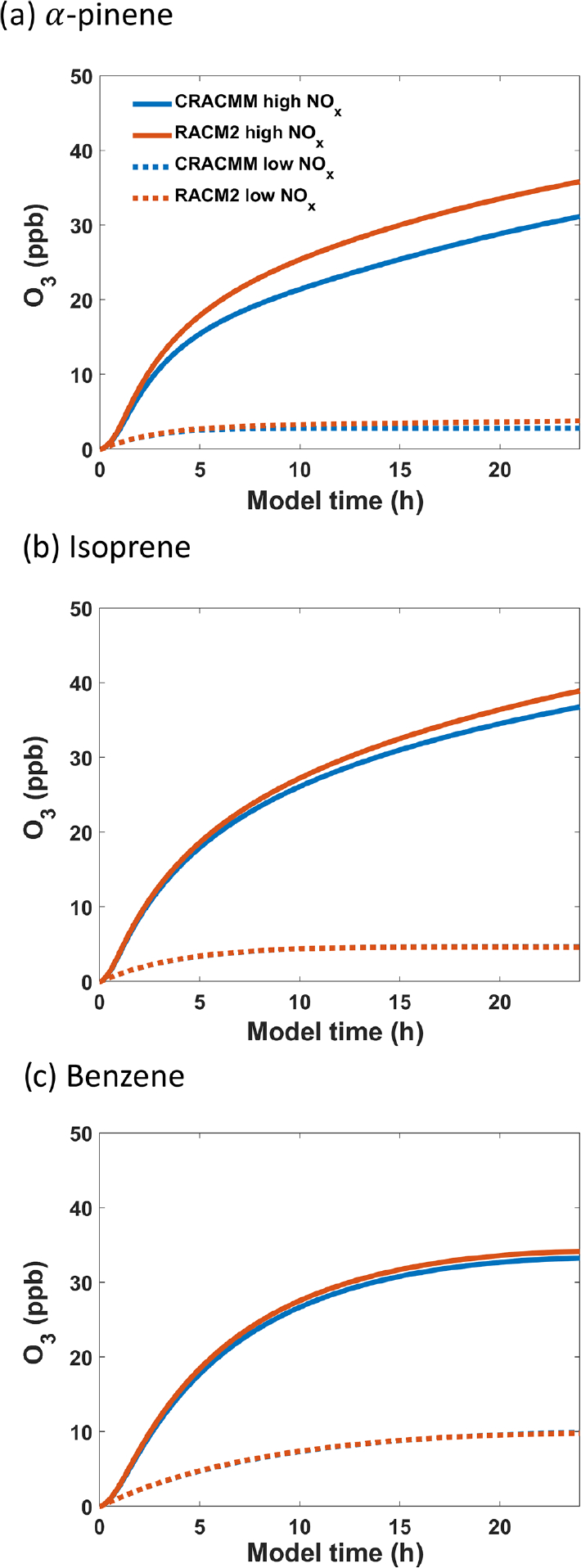
Evolution of O3 from photochemical oxidation simulations in the F0AM box model using **(a)** *α*-pinene, **(b)** isoprene, and **(c)** benzene as ROC precursors under high-NO*x* (5 ppb) and low-NO*x* (0.5 ppb) conditions.

The production of O_3_ over time predicted by RACM2 and CRACMMv1.0 under both high- and low-NO_*x*_ conditions is plotted in Fig. 6 for all three ROC precursor system simulations. The evolution of O_3_ over time followed similar trends in both mechanisms and confirms that updates made to the different ROC systems in CRACMMv1.0 did not lead to massive changes in the kinetics of ozone production. For all three high-NO_*x*_ (5 ppb) simulations, RACM2 led to higher-O_3_ predictions than CRACMMv1.0. The largest mechanism differences in O_3_ production occurred in the simulation run with *α*-pinene under higher-NO_*x*_ conditions, where 31.1 ppb of O_3_ was produced by CRACMMv1.0 vs. 35.8 ppb produced by RACM2 by the end of the simulation (Fig. 6a). The absolute difference in O_3_ production between RACM2 and CRACMMv1.0 (CRACMMv1.0 − RACM2, −3.2 ppb) in the *α*-pinene high-NO_*x*_ simulation corresponded to a relative difference of −13.1 % (Table 3). The differences in O_3_ between CRACCMv1.0 and RACM2 for the simulations run with isoprene (36.8 vs. 38.9 ppb of O_3_) and benzene (33.3 vs. 34.2 ppb of O_3_) under high-NO_*x*_ conditions were lower than those predicted for *α*-pinene (Fig. 6b, c) but still indicated mechanism differences of up to −5.7 % (Table 3). The total amount of O_3_ produced in the three simulations under low-NO_*x*_ conditions (0.5 ppb) was lower and ranged from 4.7 to 9.9 ppb (Fig. 6), with the overall changes in ozone between mechanisms very minor for the isoprene and benzene systems (O_3_ changes within 2.2 %). The largest relative changes in O_3_ production under lower-NO_*x*_ conditions (−26.3 %) between the mechanisms was again observed in the simulation initiated with *α*-pinene.

The absolute and relative differences in O_3_ production between the two mechanisms were reduced in almost all simulations when RACM2 inorganic rates were updated (RACM2_mod) to match those in CRACMMv1.0 (Table 3). The relative difference in O_3_ production in the simulations initiated with 5 ppb NO_2_ and 10 ppb ROC using RACM2_mod decreased from −13.1 % to −10.4 % in the *α*-pinene simulation, decreased from −5.7 % to −2.1 % in the isoprene simulation, and decreased from −2.6 % to −1.8 % in the benzene simulation. Further, in the low-NO_*x*_ simulations run with RACM2_mod, O_3_ differences were reduced to within 0.5 % of CRACMMv1.0 for the isoprene and benzene systems. The only simulation that showed an increase in O_3_ production when RACM2_mod was run in place of RACM2 was the simulation run with *α*-pinene under low-NO_*x*_ conditions, where relative differences in O_3_ production increased from −26.3 % to −28.2 %.

The results presented in Table 3 indicate that differences in the representation of organic chemistry in CRACMMv1.0 vs. RACM2 do partially explain the differences in O_3_ concentrations from CMAQ across the northeastern US model domain, given that mechanism differences in O_3_ production still remained in all simulations after inorganic rate constants were matched between the mechanisms. In particular, a majority of the observed O_3_ differences in the *α*-pinene–NO_*x*_–O_3_ system (≥ 80 %) under both high- and low-NO_*x*_ conditions resulted from changes to the organic reactions alone. A large fraction of the O_3_ differences (~ 70 %) in the benzene–NO_*x*_–O_3_ system were also driven by organic reaction updates for the simulations run with higher NO_*x*_. As anticipated, organic reaction change updates played a smaller role in the simulations with isoprene; however a difference in O_3_ production of 2 % still remained after running the simulations with RACM2_mod. Since RACM2_ae6 O_3_ predictions in CMAQ were shown to be generally biased high for the northeastern US (Table 1) and biogenic emissions were shown to be important for ozone formation (Fig. 4a), reductions in O_3_ production in CRACMMv1.0 contributed to the more accurate average O_3_ predictions across the northeastern US compared to RACM2_ae6. Previous work has found properly representing monoterpene chemistry, in particular, is important for accurately predicting organic nitrates and thereby ozone across North America (Browne et al., 2014; Fisher et al., 2016; Zare et al., 2018), including in the northeastern US (Schwantes et al., 2020).

Further investigation into the mechanisms revealed that there were also differences in the predicted loss of NO_*x*_ between RACM2_mod and CRACMMv1.0 (Fig. S13) and that the differences in the evolution of NO_*x*_ with time were highest in the experiment run with *α*-pinene. Thus, the parameterization of monoterpene reactions (which included the addition of autoxidation and explicit second-generation chemistry of monoterpene nitrates and aldehydes) led to both decreased O_3_ production and increased loss of NO_*x*_ in CRACMMv1.0 vs. RACM2. Despite a reduction in organic nitrate yield in the benzene system (0.2 % in CRACMMv1.0 and 8.2 % in RACM2_mod), there was also higher NO_*x*_ loss observed in the benzene simulation run with 5 ppb NO_2_ (Fig. S13). Overall, the mechanism differences in NO_*x*_ loss, in addition to ozone production, are consistent with predicted differences in OPE across the northeastern US in CRACMMv1.0 vs. RACM2 (Fig. 5).

## 5 Conclusions

This study provides the first evaluation of O_3_ predictions using the newly developed CRACMMv1.0 chemical mechanism in the context of other currently available mechanisms and demonstrates CRACMMv1.0 can provide accurate ozone predictions. Average O_3_ predictions across CRACMMv1.0, CB6r3_ae7, and RACM2_ae6 simulations during the summer of 2018 over the northeastern US were generally within ±10 % of each other, and all had domain-wide mean biases of less than 5 ppb. Mechanism differences were most pronounced over bodies of water, where meteorology amplified differences. Over land, domain-wide O_3_ estimates in CRACMMv1.0 were found to be of similar magnitude to the CMAQv5.3.3.3 operational mechanism (CB6r3_ae7) (±1 ppb) but were universally lower in the mechanism upon which CRACMMv1.0 was built (RACM2_ae6) by 1–3 ppb. The lower O_3_ concentrations and OPE in the CRACMMv1.0 simulation compared to RACM2_ae6 resulted in better predictions of all-hour and MDA8 O_3_ concentrations across the NE US region as indicated by reductions in the mean bias, normalized mean bias, and normalized mean error.

CRACMMv1.0 evaluation against AQS ozone observations indicated it is more skilled at predicting ozone in locations with elevated ozone, which is important for understanding sources of exposure at concentrations most likely to cause harm. CRACMMv1.0 showed improved performance over the current CMAQ operational mechanism (CB6r3_ae7) when hourly ozone was elevated above 50 ppb. Spatially, CRACMMv1.0 showed lower bias in the northeastern US urban corridor and higher bias at rural sites, particularly in the Appalachian Mountains. Similar results were found for diurnal predictions at individual sites where CRACMM best matched O_3_ observations at a site that experienced higher NO_*x*_ concentrations. As regional boundary conditions for CRACMMv1.0 were obtained from CB6r3_ae7, the full effects of CRACMMv1.0 on regional background air quality and long-range transport predictions have yet to be fully examined. Further, since the coupling of meteorology and chemistry has been shown to play a major role in O_3_ distributions, the robustness of the mechanism should also be tested on a variety of domains that encompass different terrains.

Improvements in CRACMMv1.0 compared to RACM2_ae6 O_3_ predictions were driven by updates to the inorganic reaction rate constants as well as updates in the representation of organic chemistry. These updates also caused slight changes in the sensitivity of ozone–ROC precursor emissions. Box model simulations in F0AM showed lower O_3_ production and higher NO_*x*_ loss for monoterpene oxidation consistent with the lower overall OPE predicted across the northeastern US with CRACMMv1.0 compared to RACM2_ae6. The zeroed emission simulations revealed that domain-wide average O_3_ estimates slightly increased when emissions of S/IVOCs were omitted, suggesting the inclusion of these emissions played a role in O_3_ formation and mainly acted to reduce ozone. As S/IVOCs are not integrated with radical chemistry leading to ozone in RACM2_ae6 or CB6r3_ae7, some changes in the sensitivity of ozone to emissions are expected in CRACMMv1.0 compared to current mechanisms. As a further example, zeroed BTX emissions indicated rural ozone is relatively insensitive to aromatic emissions in CRACMMv1.0, whereas RACM2_ae6 (and CB6r3_ae6) predicted ozone disbenefits (increases) in the rural northeastern US when aromatic emissions were removed.

Isoprene and monoterpenes, largely from biogenic sources, are examples of chemical systems where accurate representation of their chemistry across phases is critical to improve prediction of both ozone and fine-particle endpoints. As with RACM2_ae6, CRACMMv1.0 O_3_ concentrations showed great sensitivity to biogenic emissions, emphasizing the need to represent their NO_*x*_ cycling and radical chemistry well. In addition, autoxidation products with low volatility that sequester radicals are abundant from monoterpenes and critical for SOA formation (Pye et al., 2019). Separate work building on CRACMMv1.0 demonstrated that updated isoprene chemistry led to improved ozone predictions at high (> 50 ppb) concentrations as well as predictions of isoprene epoxydiol SOA precursors (Wiser et al., 2022). This need to have gas-phase mechanisms predict intermediates leading to SOA and have SOA products removed from the gas phase was a major motivation behind the development of CRACMM. Future evaluation of the fine-particle predictions of CRACMMv1.0 will provide even further constraints on the radical chemistry leading to ozone explored here.

## Supplementary Material

Supplement1

## Figures and Tables

**Table 1.** Performance of domain-wide site-specific average hourly O_3_ (number of observations, *n* = 652 476), MDA8 O_3_ (*n* = 27 037), and hourly O_3_ above 50ppb (*n* = 69 103) in terms of mean bias (MB), the Pearson correlation coefficient (*r*), normalized mean bias (NMB), and normalized mean error (NME) for the CRACMMv1.0, and CB6r3_ae7, and RACM2_ae6 simulations. The last rows reflect conditions when observed hourly ozone was above 50 ppb.

Metric	Mechanism	Domain-wide	Domain-wide	Domain-wide	Domain-wide
		MB* (ppb)	correlation (*r*)	NMB (%)	NME (%)

Hourly O_3_	CRACMMv1.0	+2.7	0.75	+8.8	27.2
	CB6r3_ae7	+2.4	0.75	+7.9	26.8
	RACM2_ae6	+4.3	0.75	+ 14.0	28.7

MDA8 O_3_	CRACMMv1.0	+2.1	0.76	+7.7	15.8
	CB6r3_ae7	+1.5	0.76	+3.4	13.5
	RACM2_ae6	+4.2	0.75	+9.6	15.9

Hourly O_3_ above 50 ppb	CRACMMv1.0	−4.7	0.54	−8.0	15.0
	CB6r3_ae7	−6.2	0.53	−10.6	15.2
	RACM2_ae6	−1.7	0.54	−2.8	14.6

* Equations used for the calculations of MB, *r*, NMB, and NME are reported in the Supplement.

**Table 2.** List of emission perturbations relative to the base simulations in CMAQ. In the case of IVOC and SVOC emission perturbations, species over a saturation concentration, C*, range are modified.

Chemical mechanism	Emission perturbation

CRACMMv1.0	Benzene-, toluene-, and xylene-like emissions set to zero

CRACMMv1.0	Biogenic-ROC emissions set to zero

CRACMMv1.0	Anthropogenic-olefin emissions set to zero

CRACMMv1.0	IVOC (C* range 10^3^–10^6^ μg m^−3^) emissions set to zero

CRACMMv1.0	SVOC (C* range 10^−2^–10^2^ μg m^−3^) emissions set to zero

CRACMMv1.0	HC10 (decane and species of similar reactivity) emissions set to zero

RACM2_ae6	Benzene-, toluene-, and xylene-like emissions set to zero

RACM2_ae6	Biogenic-ROC emissions set to zero

CB6r3_ae7	Benzene-, toluene-, and xylene-like emissions set to zero

**Table 3.** Absolute and relative differences between CRACMMvl.0 and RACM2 in the amount of ozone produced (ppb) in box model simulations run with *α*-pinene, isoprene and benzene under both low-NO_*x*_ (0.5 ppb) and high-NO_*x*_ (5 ppb) conditions. All results are reported relative to CRACMMv1.0.

ROC precursor	Chemical mechanism difference	Absolute difference in O_3_ (high NO_*x*_)	Relative difference in O_3_ (high NO_*x*_)	Absolute difference in O_3_ (low NO_*x*_)	Relative difference in O_3_ (low NO_*x*_)

*α*-Pinene	CRACMMv1.0–RACM2	−4.7 ppb	−13.1%	−1.0 ppb	−26.3%
Isoprene	CRACMMv1.0–RACM2	− 2.1 ppb	−5.7%	+0.1 ppb	+2.2%
Benzene	CRACMMv1.0–RACM2	−0.9 ppb	−2.6%	+0.1 ppb	+1.0%
*α*-Pinene	CRACMMv1.0–RACM2_mod	−3.6 ppb	−10.4%	−1.1 ppb	−28.2%
Isoprene	CRACMMv1.0–RACM2_mod	−0.8 ppb	−2.1 %	< 0.1 ppb	< 0.5 %
Benzene	CRACMMv1.0–RACM2_mod	− 0.6 ppb	−1.8%	< 0.1 ppb	< 0.5%

## Footnotes

**Code and data availability.** The implementation of RACM2_ae6 and CB6r3_ae7 used here is available in CMAQv5.3.3 (https://doi.org/10.5281/zenodo.5213949, U.S. Environmental Protection Agency Office of Research and Development, 2021). CRACMMv1.0 is available in CMAQv5.4 (https://doi.org/10.5281/zenodo.7218076, U.S. EPA Office of Research and Development, 2022). Supporting data for CRACMM including guidance on emission preparation and species metadata (including SMILES identifiers; simplified molecular-input line-entry system) are available at https://github.com/USEPA/CRACMM (U.S. Environmental Protection Agency, 2022c and in the work of Pye et al., 2023). AMET is available at https://github.com/USEPA/AMET (U.S. Environmental Protection Agency, 2022d) and https://doi.org/10.5281/zenodo.8156171 (Appel and Gilliam, 2023). F0AM is available at https://github.com/AirChem/F0AM (Wolfe, 2022). Specific analyses and scripts used in this paper, such as the modeled and observed ozone concentrations, F0AM box model inputs, and exact CMAQ code used, are archived at https://doi.org/10.23719/1528552 (Pye, 2023).
