# Supplementary material for: Sensitivity of northeastern US surface ozone predictions to the representation of atmospheric chemistry in the Community Regional Atmospheric Chemistry Multiphase Mechanism (CRACMMv1.0)
Source: Atmos Chem Phys. Author manuscript; Available in PMC 2024 Oct 21. (PMC11492977; doi:10.5194/acp-23-9173-2023)
Supplement: Supplement1 [file NIHMS2020162-supplement-Supplement1.pdf]

Supplement of Atmos. Chem. Phys., 23, 9173–9190, 2023
https://doi.org/10.5194/acp-23-9173-2023-supplement
© Author(s) 2023. CC BY 4.0 License.

Atmospheric Chemistry and Physics
Open Access
EGU

Supplement of

Sensitivity of northeastern US surface ozone predictions to the representation of atmospheric chemistry in the Community Regional Atmospheric Chemistry Multiphase Mechanism (CRACMMv1.0)

Bryan K. Place et al.

Correspondence to: Havala O. T. Pye (pye.havala@epa.gov)

The copyright of individual parts of the supplement might differ from the article licence.

Equations used for calculating site-specific statistics reported in Table S1

Equation 1. $$MB = \frac{1}{S}\sum_{j=1}^{S}\left[\frac{1}{T}\sum_{i=1}^{T}\left(p_{i,j} - o_{i,j}\right)\right]$$

Equation 2. $$r = \frac{1}{S}\sum_{j=1}^{S}\left[\frac{\sum_{i=1}^{T}(p_{i,j}-\overline{p_J})(o_{i,j}-\overline{o_J})}{\sum_{i=1}^{T}(p_{i,j}-\overline{p_J})^2\sum_{i=1}^{T}(o_i-\overline{o_J})^2}\right]$$

Equation 3. $$NMB = 100\% \times \frac{1}{S}\sum_{j=1}^{S}\left[\frac{\sum_{i=1}^{T}(p_{i,j}-o_{i,j})}{\sum_{i=1}^{T}(o_{i,j})}\right]$$

Equation 4. $$NME = 100\% \times \frac{1}{S}\sum_{j=1}^{S}\left[\frac{\sum_{i=1}^{T}(|p_{i,j}-o_{i,j}|)}{\sum_{i=1}^{T}(o_{i,j})}\right]$$

Where S is the number of sites, T is the number of time periods (number of 1-hour or MDA8 values), $p_{i,j}$ and $o_{i,j}$ are the model predicted and observed values over a specific time period, *i*, at site, *j*, and $\overline{p_J}$ and $\overline{o_J}$ are the mean predicted and observed values over all times at a site, *j*.

Table S1: Mapping from CRACMMv0.21 to CRACMMv1.0 and RACM2_ae6 emissions for use in CMAQ. The mechanism number changed from v0.21 (an interim version used to create CMAQ-ready CRACMM emissions) to v1.0 in the final version used in the study. RACM2_ae6 mappings are approximate based on the major contributors in each CRACMM category and information from William P.L. Carter (https://intra.engr.ucr.edu/~carter/emitdb/).

**CRACMMv0.21 species**	**CRACMMv1.0 species**	**RACM2_ae6 Species**
ACRO	ACRO	MACR
BDE13	BDE13	DIEN
BEN	BEN	BENZENE
FURAN	FURAN	HC8
HC8	HC10	HC8
NAPH	NAPH	XYM
PROG	PROG	HC8
TOLUENE	TOL	TOL
XYOP	XYE	XYO*0.5+XYP*0.5
ROCIOXY	VROCIOXY	NA

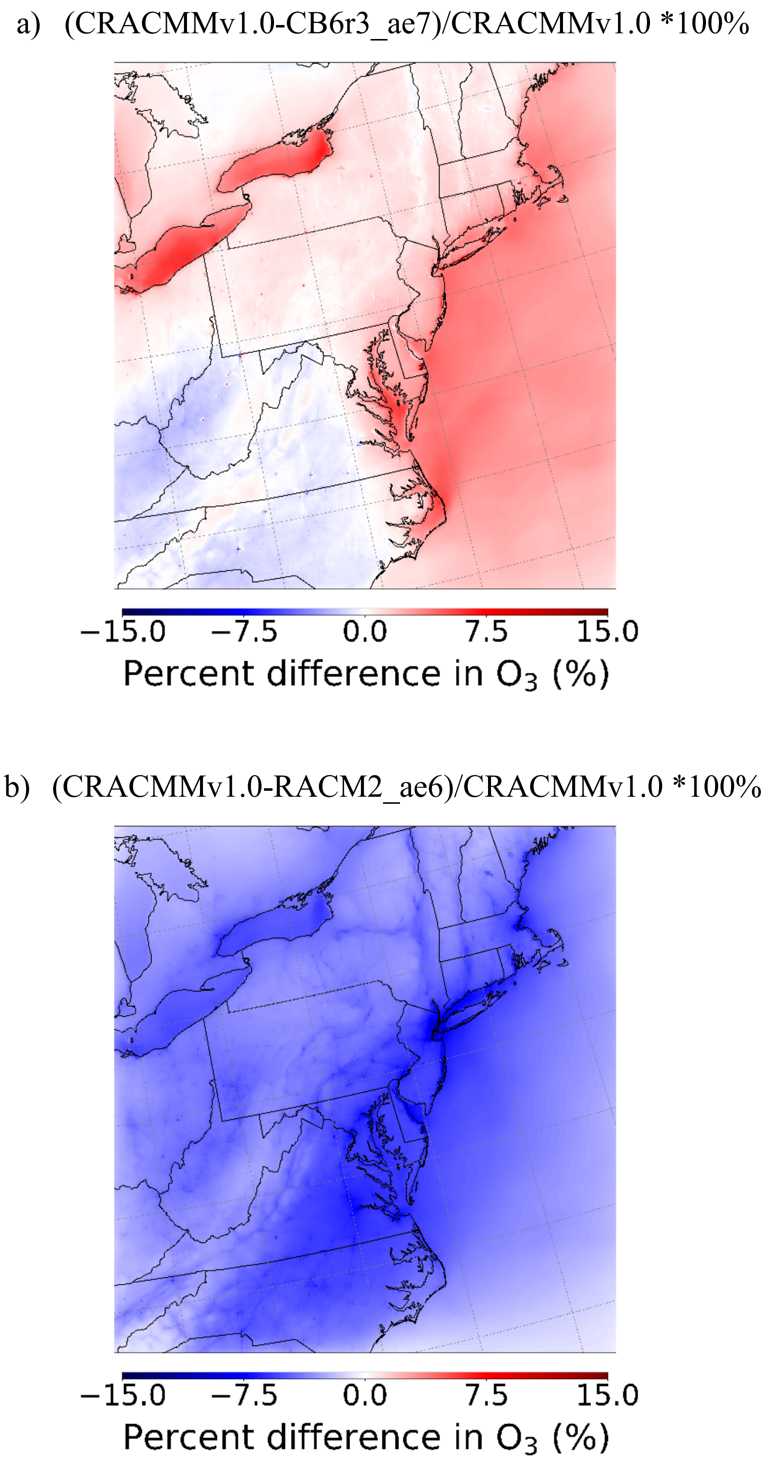

Figure S1. Percent difference in average O_3 between a) CRACMMv1.0 and Cb6r3_ae7 and b) CRACMMv1.0 and RACM2_ae6.

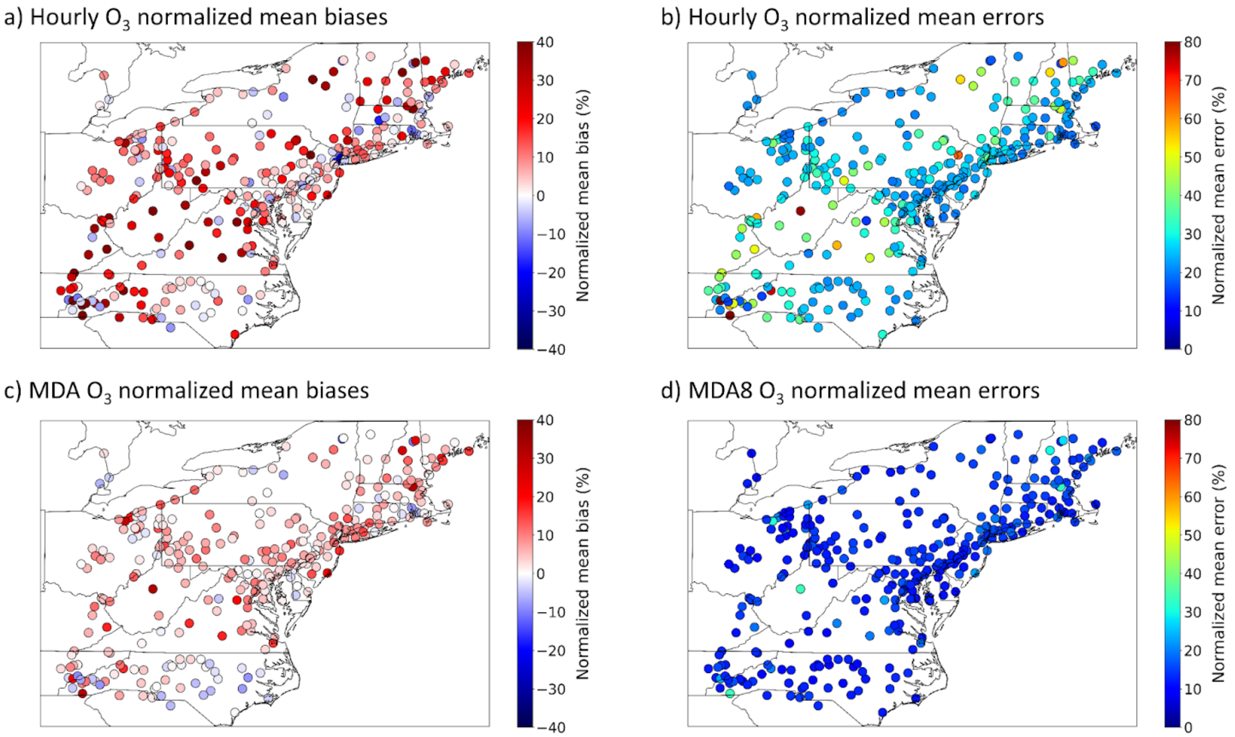

Figure S2. CRACMM hourly and MDA8 O_3 NMB and NME site statistics.

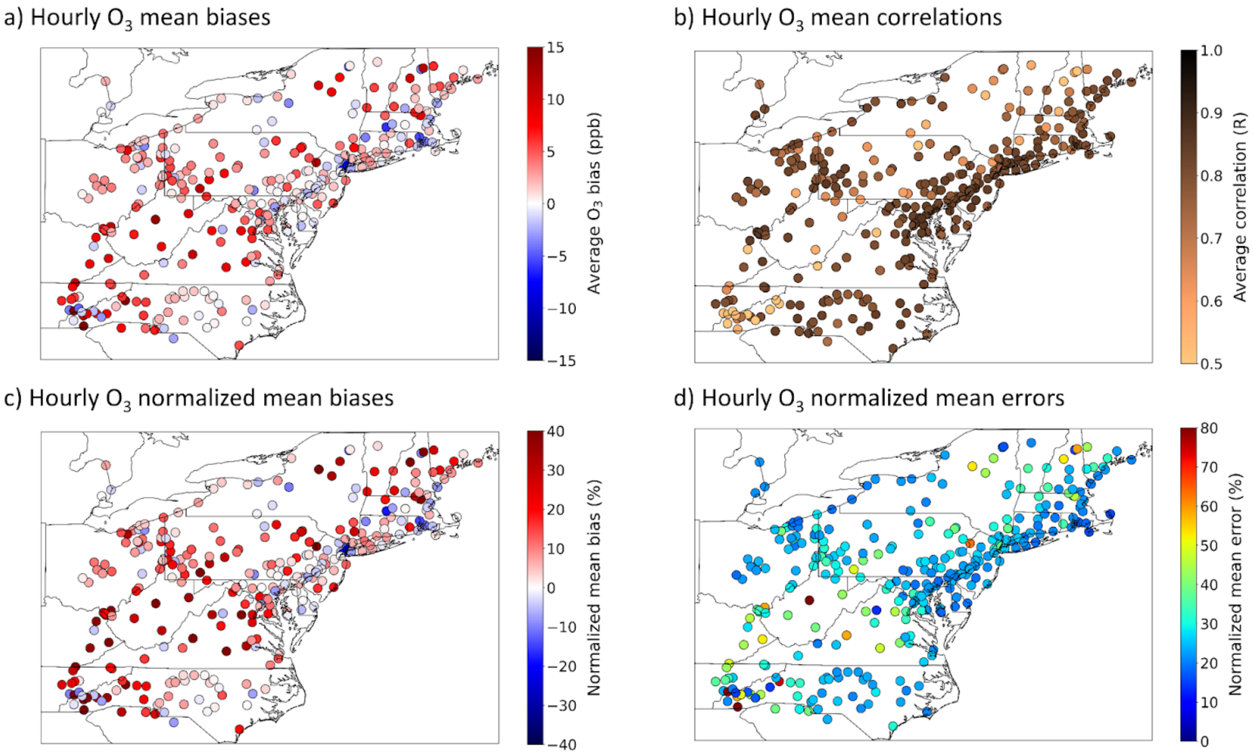

Figure S3. CB6r3_ae7 all hour site statistics.

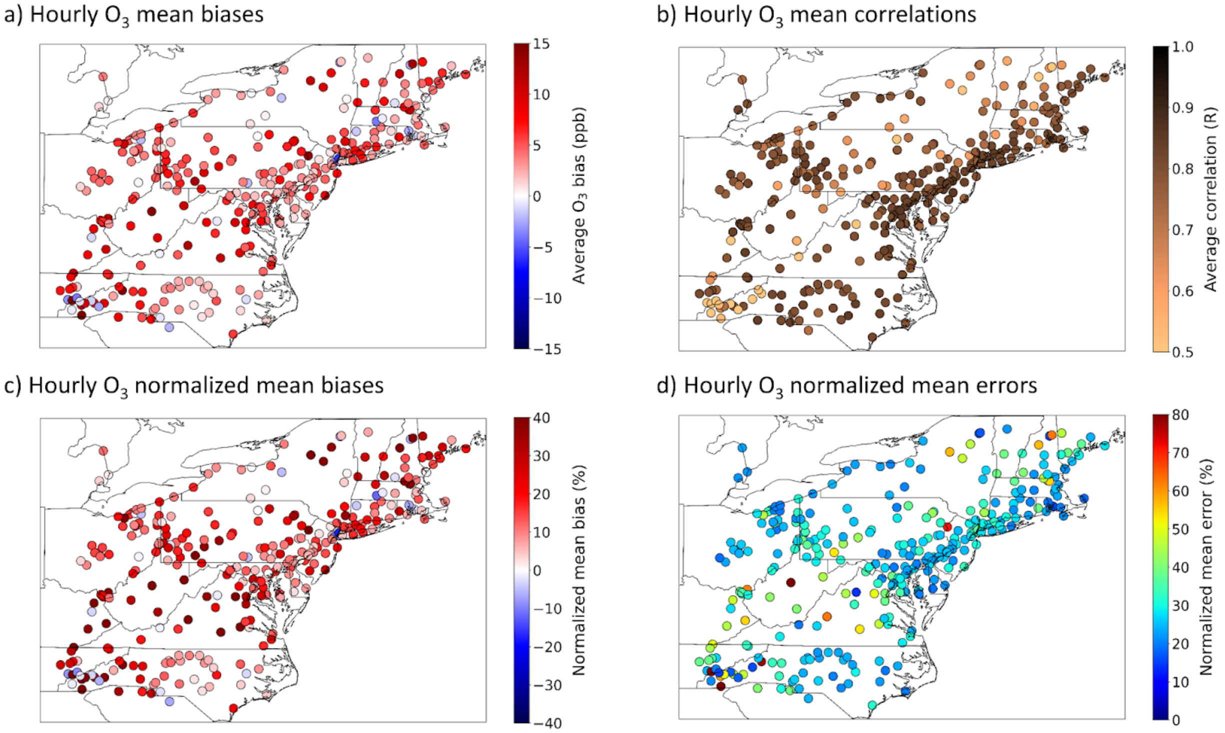

Figure S4. RACM2_ae6 all hour O_3 site statistics.

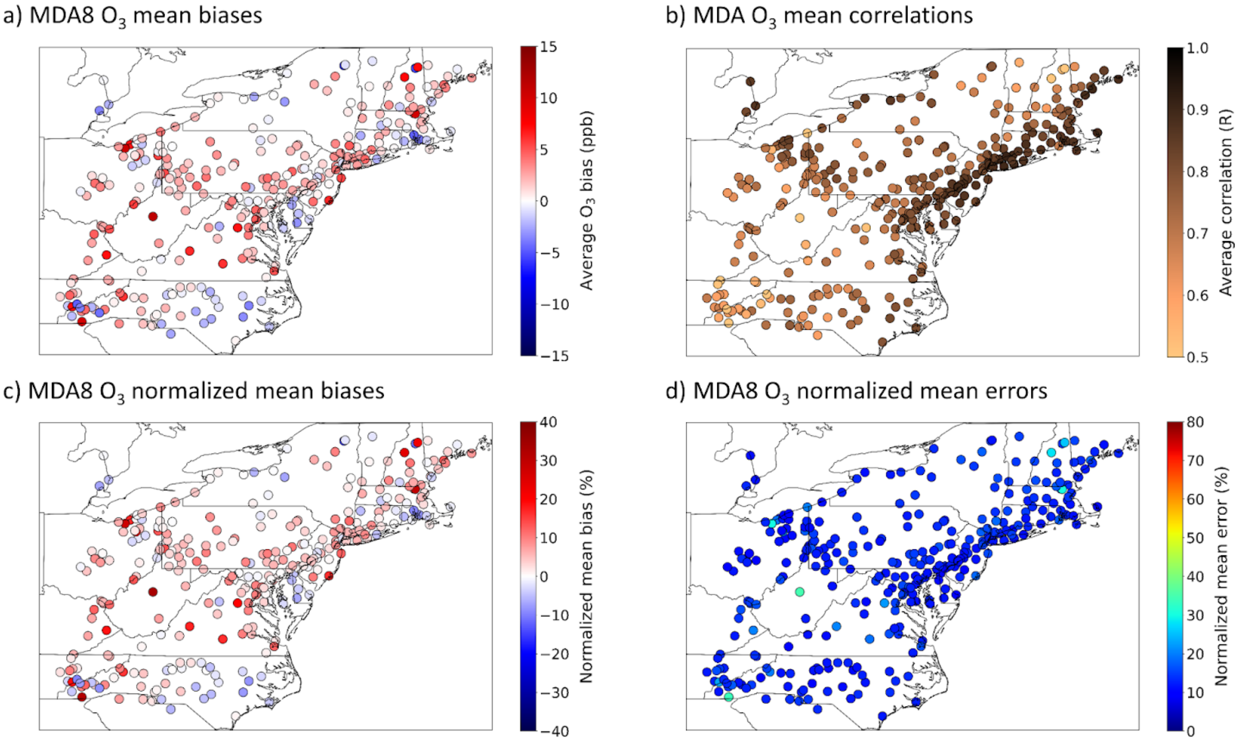

Figure S5. CB6r3_ae7 MDA8 O_3 site statistics.

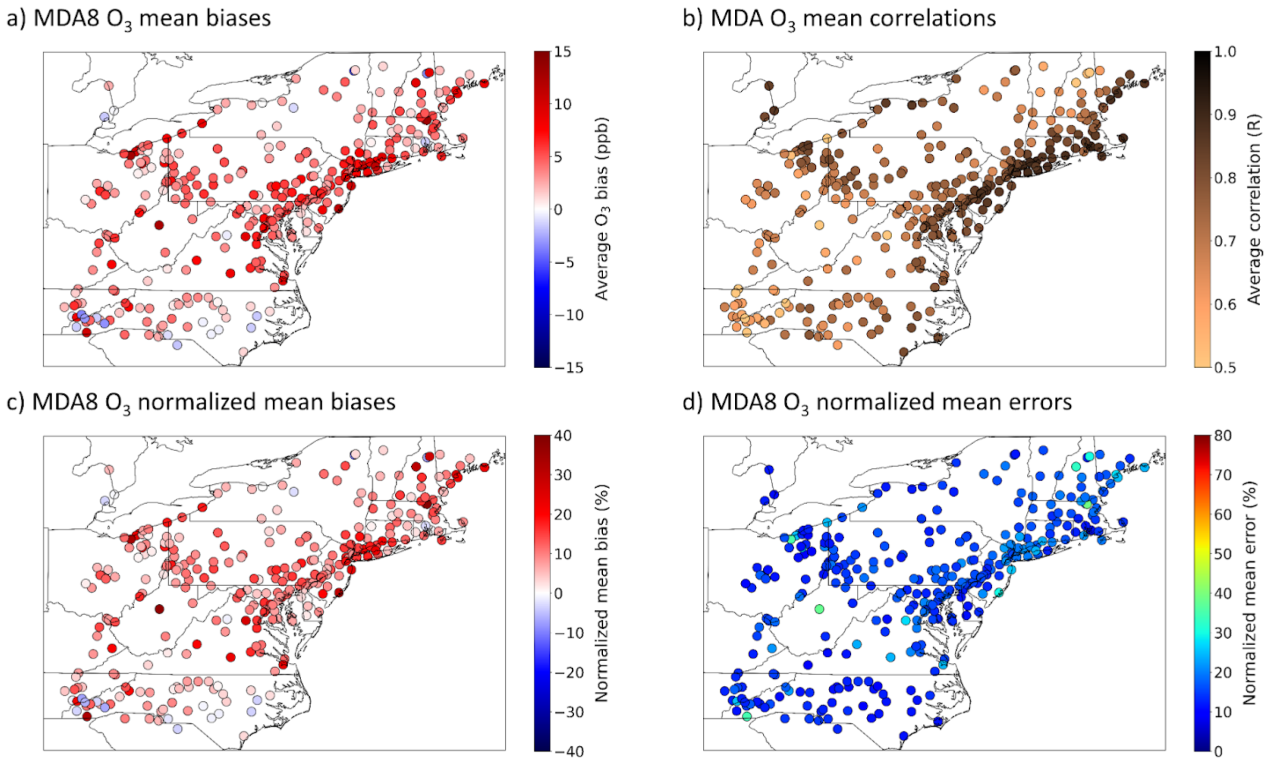

Figure S6. RACM2_ae6 MDA8 O_3 site statistics.

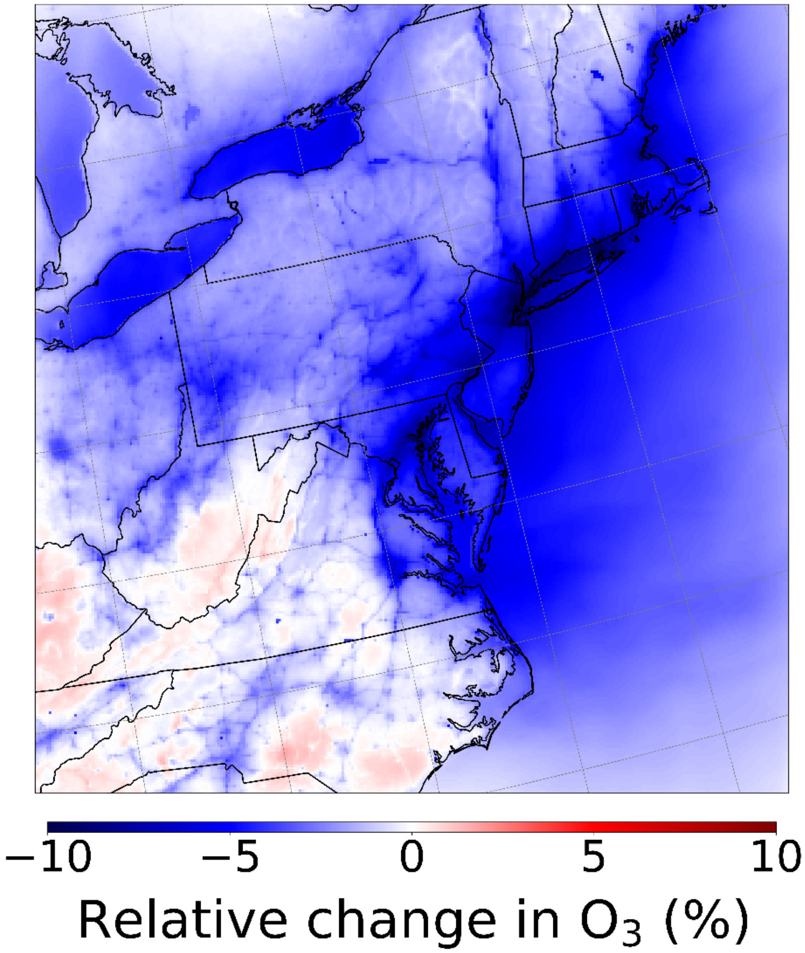

Figure S7. RACM2_ae6 biogenic zero-out percent change in O_3.

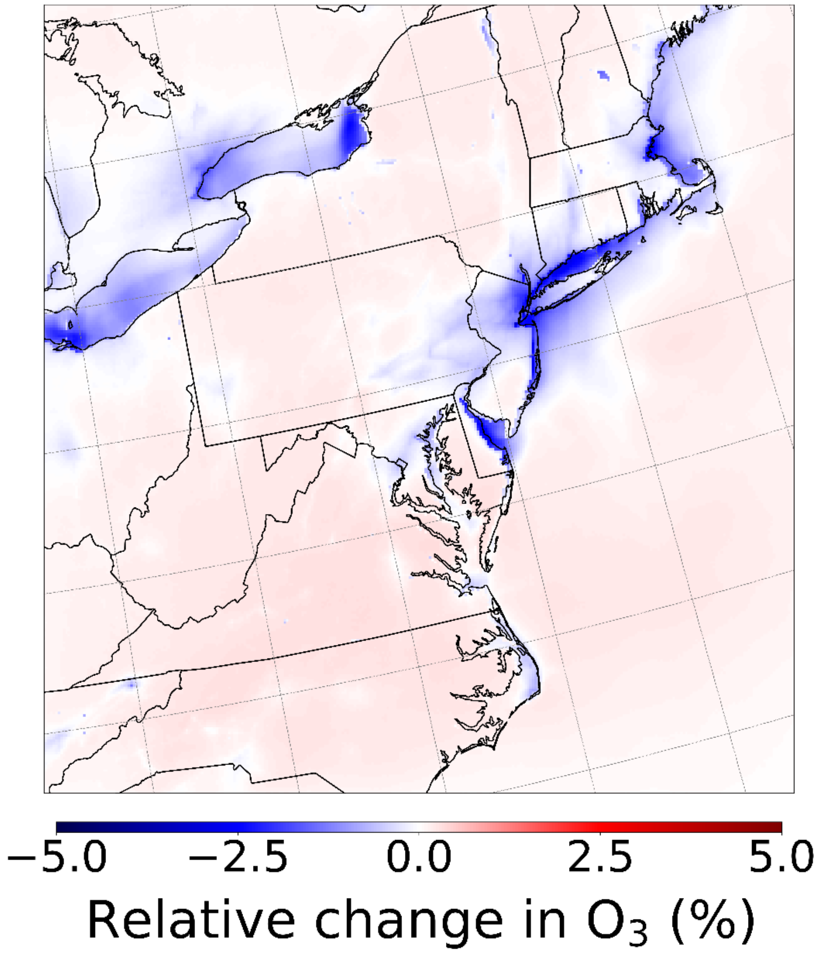

Figure S8. RACM2_ae6 BTX zero out percent change in O_3.

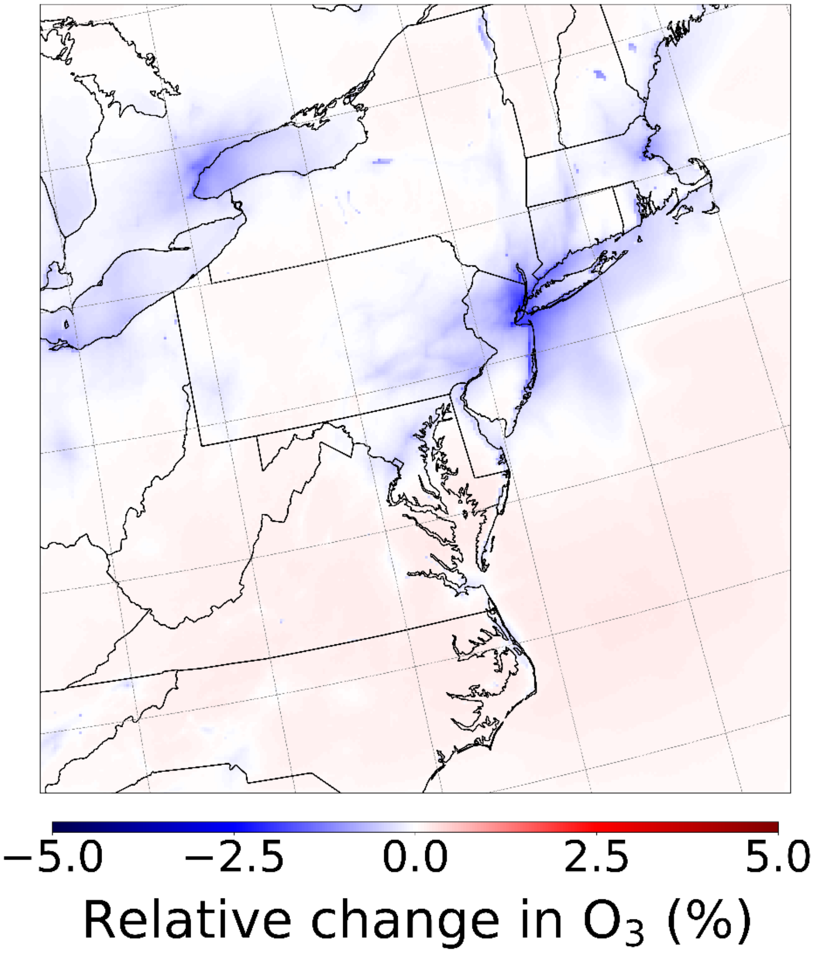

Figure S9. CB6r3_ae7 BTX zero out percent change in O_3.

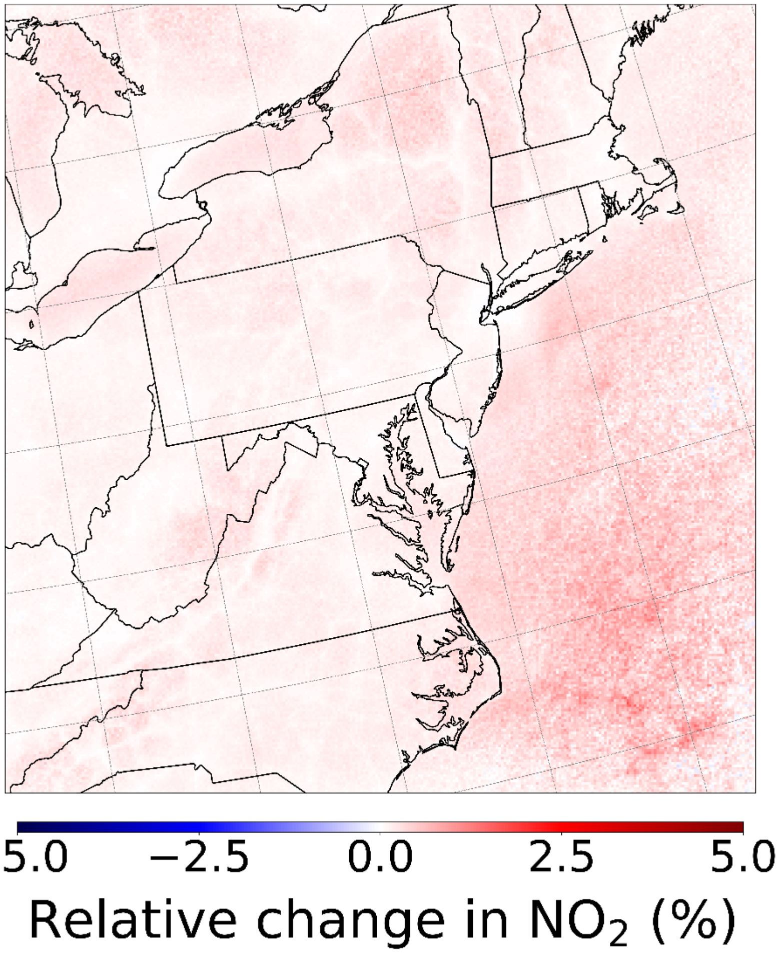

Figure S10. CRACMM HC10 zero out percent change in NO_2.

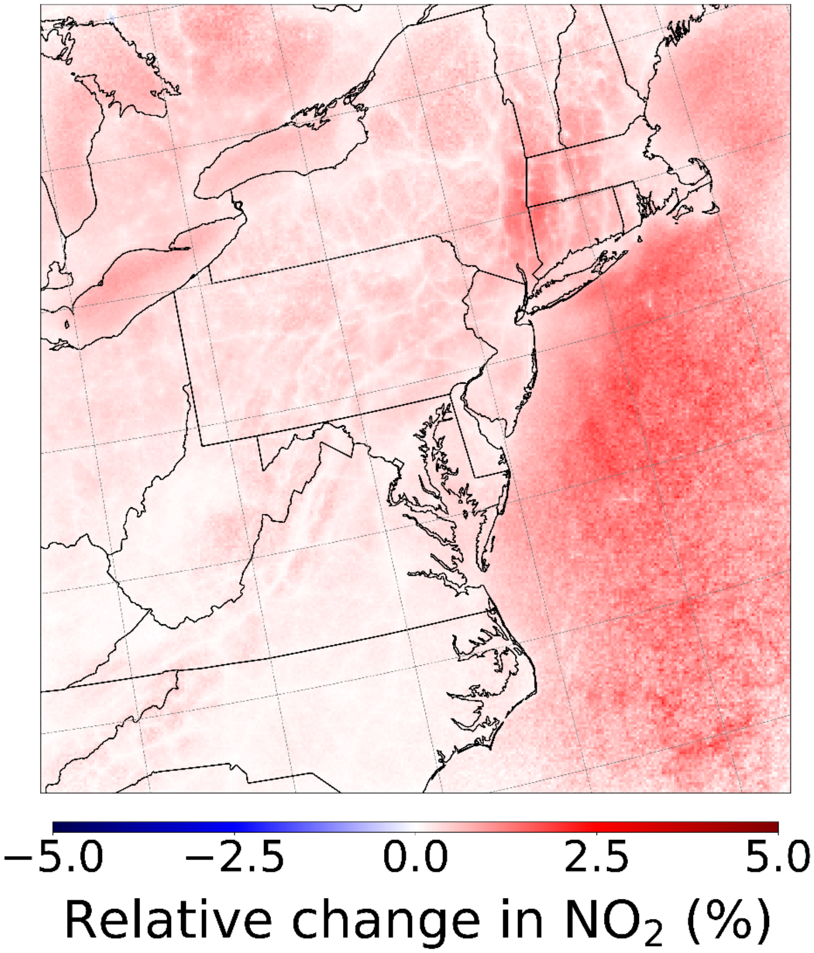

Figure S11. CRACMM SVOC zero out percent change in NO_2.

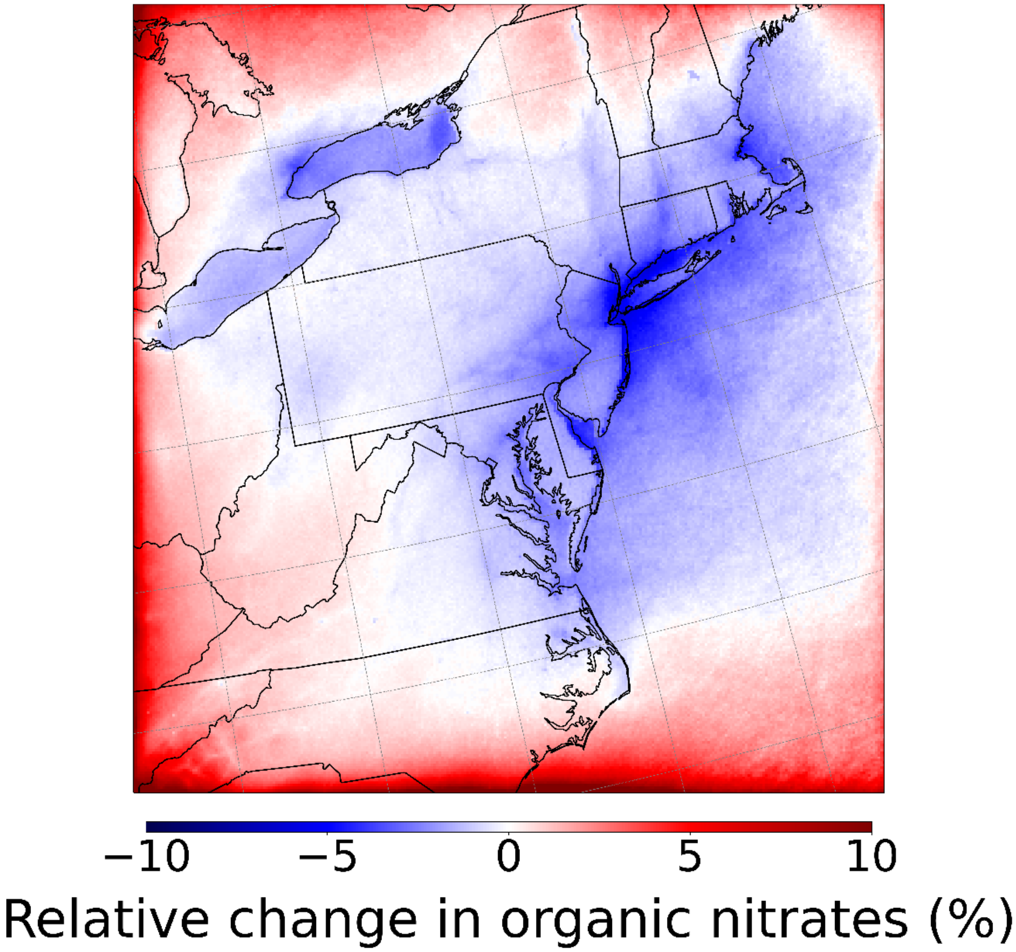

Figure S12. CRACMM HC10 zero out percent change in organic nitrates.

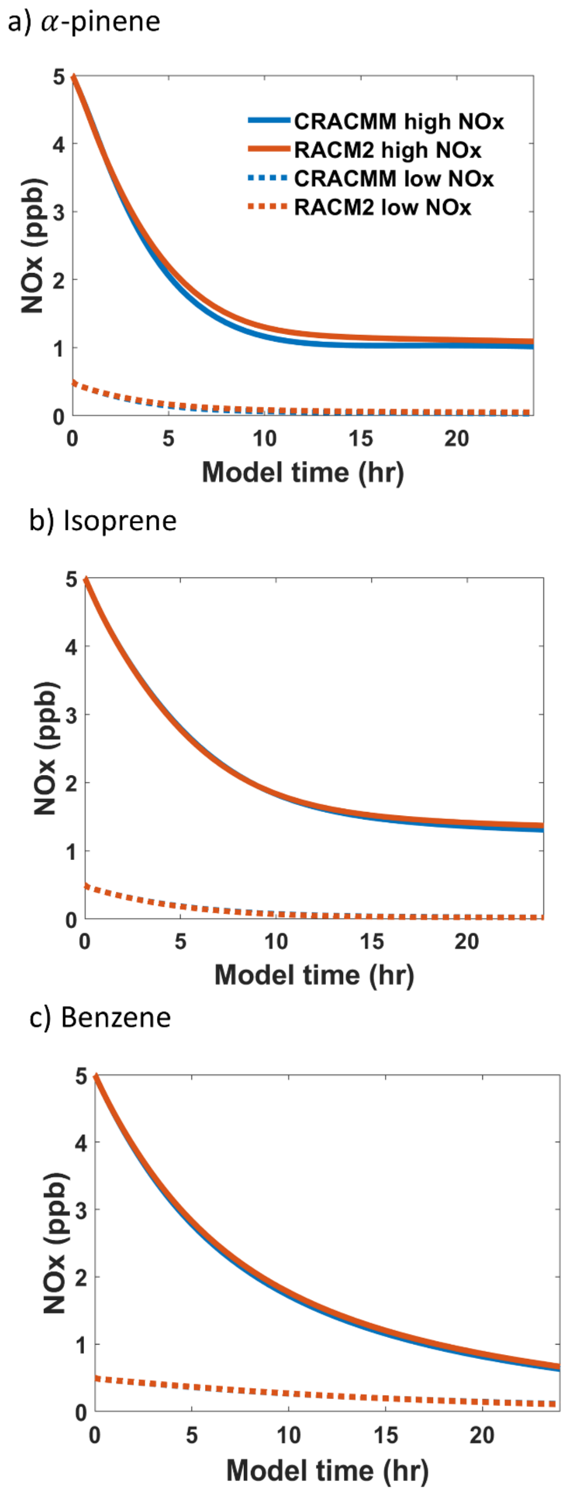

Figure S13. Evolution of NO_x from photochemical oxidation simulations in the F0AM box model simulations using a) α-pinene and b) isoprene and c) benzene as ROC precursors under high NO_x (5ppb) and low NO_x (0.5 ppb) conditions.
